# Prediction of PD-L1 inhibition effects for HIV-infected individuals

**DOI:** 10.1371/journal.pcbi.1007401

**Published:** 2019-11-06

**Authors:** Valerya Zheltkova, Jordi Argilaguet, Cristina Peligero, Gennady Bocharov, Andreas Meyerhans

**Affiliations:** 1 Faculty of Computational Mathematics and Cybernetics, Lomonosov Moscow State University, Moscow, Russia; 2 Marchuk Institute of Numerical Mathematics, Russian Academy of Sciences, Moscow, Russia; 3 Infection Biology Laboratory, Department of Experimental and Health Sciences, Universitat Pompeu Fabra, Barcelona, Spain; 4 Sechenov First Moscow State Medical University, Moscow, Russia; 5 ICREA, Pg. Lluís Companys 23, Barcelona, Spain; ETH Zurich, SWITZERLAND

## Abstract

The novel therapies with immune checkpoint inhibitors hold great promises for patients with chronic virus infections and cancers. This is based mainly on the partial reversal of the exhausted phenotype of antigen-specific cytotoxic CD8 T cells (CTL). Recently, we have shown that the restoration of HIV-specific T cell function depends on the HIV infection stage of an infected individual. Here we aimed to answer two fundamental questions: (i) Can one estimate growth parameters for the HIV-specific proliferative responsiveness upon PD-L1 blockade ex vivo? (ii) Can one use these parameter estimates to predict clinical benefit for HIV-infected individuals displaying diverse infection phenotypes? To answer these questions, we first analyzed HIV-1 Gag-specific CD8 T cell proliferation by time-resolved CFSE assays and estimated the effect of PD-L1 blockade on division and death rates, and specific precursor frequencies. These values were then incorporated into a model for CTL-mediated HIV control and the effects on CTL frequencies, viral loads and CD4 T cell counts were predicted for different infection phenotypes. The biggest absolute increase in CD4 T cell counts was in the group of slow progressors while the strongest reduction in virus loads was observed in progressor patients. These results suggest a significant clinical benefit only for a subgroup of HIV-infected individuals. However, as PD1 is a marker of lymphocyte activation and expressed on several lymphocyte subsets including also CD4 T cells and B cells, we subsequently examined the multiple effects of anti-PD-L1 blockade beyond those on CD8 T cells. This extended model then predicts that the net effect on HIV load and CD4 T cell number depends on the interplay between positive and negative effects of lymphocyte subset activation. For a physiologically relevant range of affected model parameters, PD-L1 blockade is likely to be overall beneficial for HIV-infected individuals.

## Author summary

Immune checkpoint inhibitors can revitalize exhausted virus-specific CD8 T cells (CTL) and thus hold great promises for patients with chronic virus infections. Based on the hypothesis that gain in the proliferation of CTL by such interventions may positively influence HIV-1 disease progression, we developed a general framework of how to quantify the proliferative CTL responsiveness after PD-L1 blockade, and translate it to patient outcome predictions. However, PD-L1 blockade also activates (i) exhausted CD4 T cells generating additional HIV target cells for virus expansion as well as (ii) exhausted B cells producing increasing antibody levels that potentially inhibit HIV spread. To assess these virus-specific effects of a PD-L1 blockade, we then extended our basic model and incorporated experimentally determined CD4 T cell proliferation gains and estimates on HIV-specific antibody increases. Altogether, the blockade effect is predicted to be infection phenotype-dependent and in most cases beneficial. Our general approach can be expanded to other immunotherapies and is an important step forward towards personalized treatment strategies in infectious diseases and cancers.

## Introduction

A hallmark of chronic virus infections with persisting antigen and cancer growth is the downregulation of immune effector mechanisms [1–3]. Amongst these are T cell exhaustion by deletion [4] and functional impairment [5]. The latter reflects a T cell differentiation state with defined epigenetic modifications that is characterized by surface expression of several inhibitory receptors like PD-1, TIM-3, LAG-3 and TIGIT, and impaired proliferative responsiveness and effector cytokine secretion upon antigenic stimulation [6]. Interestingly, T cell exhaustion is partly reversible by antibodies that inhibit the interaction of the inhibitory receptors with their ligands [2]. This exhausted T cell (Tex) revitalization, albeit transient, can lead to a reduction of virus titers and cancer growth [7]. A respective immunotherapeutic strategy, also known as immune checkpoint inhibitor therapy, has been used for the treatment of late-stage cancers with significant benefit for some of the patients [8]. A search for reliable predictive biomarker that could identify therapy-responsive patients is presently pursued with intensity.

There is early evidence that Tex revitalization can reduce virus loads in chronic lymphocytic choriomeningitis virus (LCMV)-infected mice [5] and simian immunodeficiency virus (SIV)-infected monkeys [9]. Importantly, the PD-1 blockade of SIV251-infected monkeys not only led to rapid expansion of virus-specific CD8 T cells and virus load reduction, but also to prolonged animal survival thus providing strong evidence for a potential benefit of checkpoint inhibitors in HIV-infected humans. However, most clinical trials with checkpoint inhibitors were performed with cancer patients and excluded patients with chronic infections. Thus, data about the effects of these therapies in virus infections in humans are scarce. Nonetheless, the available data suggest that anti-PD-1 or anti-PD-L1 treatments are safe for HIV-infected individuals, and that their viral loads did not augment in meaningful ways while on antiretroviral treatment [10]. Results from larger trials that are currently performed mainly in HIV patients with cancers are urgently needed to evaluate which patients will eventually have long-term therapy benefits [11].

**Fig 1:**
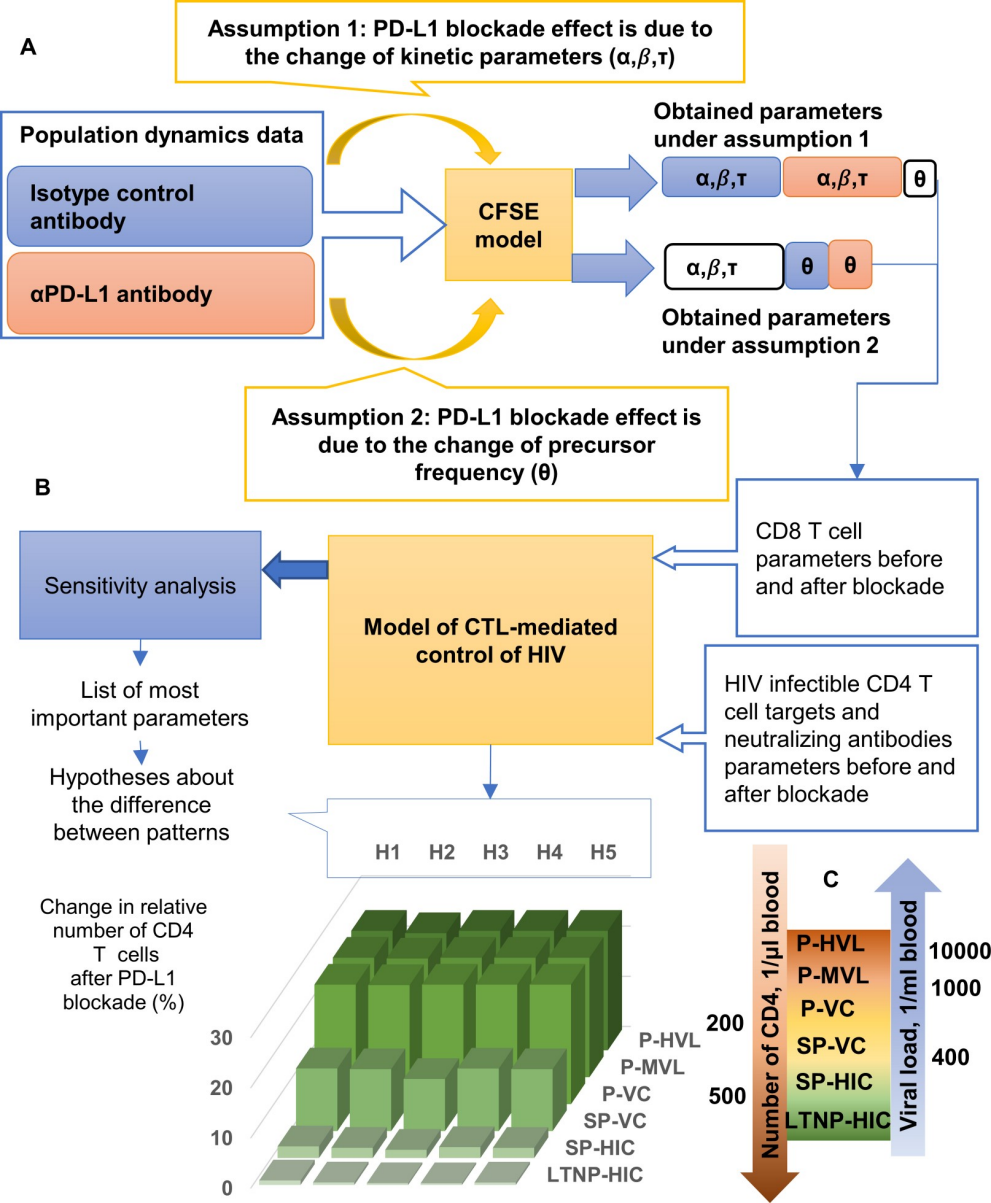
Scheme for the model-based analysis of the effect of PD-L1 blockade on CD4 T cell and virus load quasi-steady states for diverse HIV infection phenotypes. (A) Analysis scheme for HIV-specific CD8 T cell proliferative responsiveness towards PD-L1 blockade. Two assumptions are implemented to estimate separately the effect on turnover rates and change of specific T cell precursor frequencies. (B) Prediction scheme for the effect of the PD1-induced proliferative responsiveness on different HIV phenotypes based on a model for CTL-mediated HIV control. HIV phenotypes are based on CD4 T cell counts, virus load and progression rates [18]. For each phenotype, a range of underlying mechanisms is hypothesized and connected to the model parameters virus elimination rate c (H1), total number of virus particles produced per infected cell N (H2), the total influx of un-infected CD4 T cells s (H3) and the infection rate k (H4). Hypothesis 5 (H5) considers s and c. (C) Definition of HIV infection phenotypes [18]. P-HVL, progressors with high viral load; P-MVL, progressors with medium viral load, P-VC, progressors, viral controllers; SP-VC, slow progressors, viral controllers; SP-HIC, slow progressors, HIV controllers; LTNP-HIC, long-term nonprogressors, HIV controllers. The predicted change in CD4 T cell counts for the range of phenotypes and different hypotheses is shown in green as an example based on the proliferative responsiveness of donor 156.

Checkpoint inhibitors increase proliferation and function of exhausted T cells, and HIV-specific T cell proliferation is directly linked with low viremia and control of HIV disease progression [12–17]. Based on these observations we hypothesized that the gain of CD8 T cell proliferation in the presence of PD-1 blockade should positively influence HIV disease progression. In the present study, we first measured the HIV-1 Gag-specific proliferative responsiveness of CD8 T cells from HIV-infected individuals by CFSE-based flow cytometry, calculated dynamic parameter values from time-series measurements and used the estimated values to predict potential outcomes for infected patients. Subsequently, we extended this basic model and incorporated (i) experimental data on HIV-specific CD4 T cell proliferative responsiveness and (ii) estimates on B cell produced HIV-neutralizing antibody increases. The overall strategy is schematically shown in Fig 1. Our results predict that PD-L1 blockade therapies should positively impact viremia decline and CD4 T cell increase in patients dependent on their status at therapy initiation and their lymphocyte subpopulation responses.

## Results

### Estimation of the gain in CD8 T cell proliferation rate under PD-L1 blockade

PD-L1 blockade increases the proliferative responsiveness of HIV-specific CD4 and CD8 T lymphocytes from HIV-infected patients ex vivo [19]. To quantify this gain of function, peripheral blood mononuclear cells (PBMCs) from HIV-1 seropositive donors were isolated from blood by Ficoll gradient, labelled with CFSE and stimulated with a pool of overlapping peptides of HIV-1 Gag in the presence and absence of PD-L1-blocking antibodies as previously described [19]. The time-series CFSE histograms for the CD8 and CD4 T cells of the five patients analyzed are shown in S1A and S1B Fig, respectively. Since the fluorescent dye CFSE within labelled cells is approximately evenly partitioned during cell divisions, the dye provides a useful surrogate for the number of divisions a cell has undergone [20–22]. Therefore, time-series CFSE histograms can be used to estimate the quantitative difference in the parameters determining the cell population growth with and without PD-L1 blockage. We estimated these parameters for HIV-specific T cells of the 5 patients by (i) decomposing the CFSE histograms into superposition of cell population cohorts that differ in terms of the number of the completed divisions and (ii) formulating a parsimonious mathematical model to describe the evolution of the cell generation with time.

#### Decomposition of CFSE histograms

To decompose the CFSE histograms, we approximated the histogram time series data ${\left(t_{k},H_{k}\right)}_{k=1}^{K}$ by a sum of the Gaussian functions $G_{i}(x)=\frac{1}{\sigma_{i}\sqrt{2\pi }}e^{-\frac{{\left(x-\mu_{i}\right)}^{2}}{2\sigma_{i}^{2}}}$, where *i*,*μ*_*i*_,*σ*_*i*_,*x* refer to the cell cohort number (*i—*ranging from 1 to 5), the mean CFSE fluorescence of the cohort, the standard deviation of the fluorescence of the cohort and the log-transformed CFSE amount, respectively. The total number of cells in the *i*-th generation is given by $N_{i}=\int_{x_{\min}}^{x_{\max}}G_{i}(x)dx$. The cohort-specific parameters were estimated iteratively by sequentially adding one CFSE histogram at each iteration step:

$$\begin{array}{l} \mathrm{Step}\;1.k=1,\mathrm{Estimate}\;\mu_{1},\sigma_{1}\;\mathrm{by}\;\mathrm{fitting}\;\mathrm{the}\;\left(t_{k},H_{k}\right)\;\mathrm{data}\\ \int_{x_{\min}}^{x_{\max}}\left|H_{1}-\sum_{i=1}^{k}w_{i}G_{i}\right|dx\Rightarrow \underset{\mu_{1},\sigma_{1},w_{1}}{\min}\\ \mathrm{Step}\;2.k=k+1,{\hat{\mu }}_{1}^{k}=\mu_{1}^{k-1},\\ \mathrm{for}\;i\geq 2,\;\mathrm{set}\;\mathrm{initial}\;\mathrm{guess}\\ {\hat{\mu }}_{i}^{k}={\hat{\mu }}_{i}^{k}-\log_{10}2\cdot \left(i-1\right),{\hat{\sigma }}_{i}^{k}=\sigma_{i}^{k-1},\;{\hat{w}}_{i}^{k}=w_{i}^{k-1}\\ \mathrm{solve}\int_{x_{\min}}^{x_{\max}}\left|H_{k}-\sum_{i=1}^{k}w_{i}G_{i}\right|dx\Rightarrow \underset{\mu_{i},\sigma_{i},w_{i}}{\min}\\ \mathrm{Step}\;3.\mathrm{If}\;k\leq 5\;\mathrm{goto}\;\mathrm{Step}\;2\;\mathrm{else}\;\mathrm{Stop}. \end{array}$$

**Fig 2:**
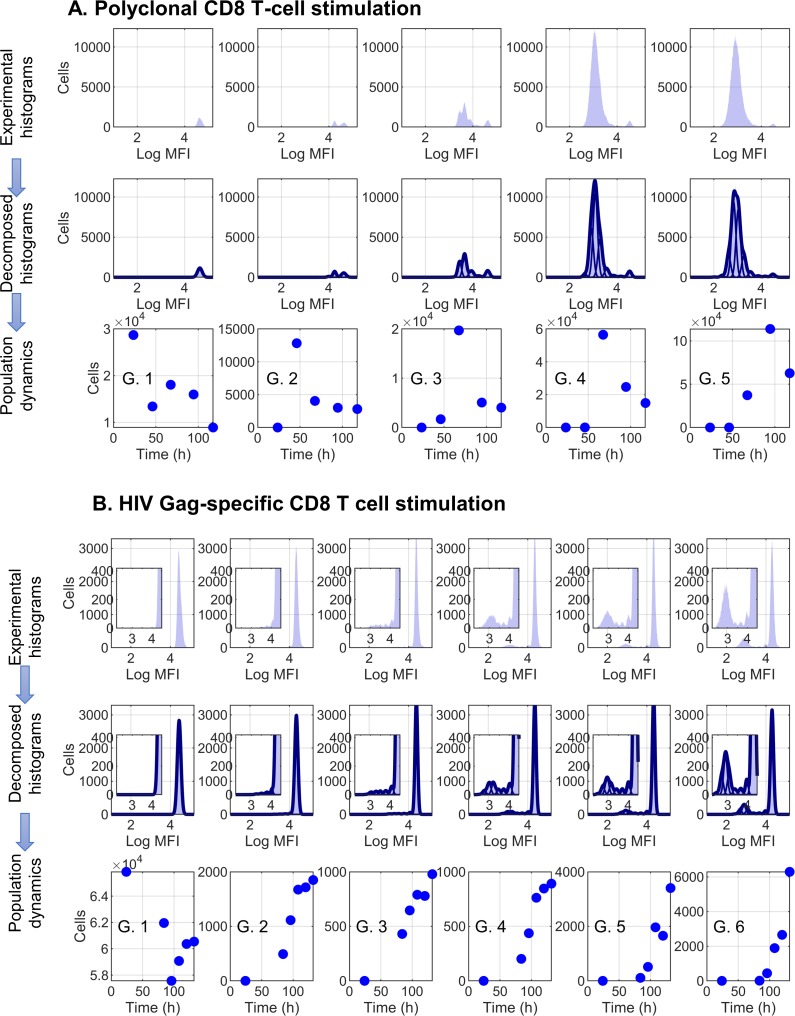
Mathematical analyses of CFSE-based proliferation assays. CD8 T cells were stimulated by Phytohaemagglutinin (PHA) (A) or HIV-1 Gag peptides (B). The experimentally determined time-series CFSE histograms, decomposed histograms and the estimated cell population sizes for the different generations are shown. Histograms were approximated by the sum of gaussians, corresponding to the generations of cells. MFI, median fluorescence intensity; G, generation.

The performance of the above algorithm for decomposition of polyclonally phytohemagglutinin (PHA)-stimulated CD8 T cells is shown in Fig 2A. The first row shows the original CFSE histograms, the second row their Gaussian-based approximations and the third row the estimated number of cells in generation 1 to generation 5. The decomposition of CFSE histograms characterizing the proliferation of HIV Gag-specific CD8 T cells is shown in Fig 2B, with the last row presenting the quantified dynamics of cell generations from 24 to 132 hours post stimulation.

#### CFSE-labeled cell proliferation model

The time-series data on cell cohorts representing different generations can now be used to evaluate the kinetic parameters of the mathematical model of T lymphocyte growth following polyclonal or antigen-specific stimulation. Mathematical models of cell growth can take various forms and differ in their complexity depending on the parameters of interest and the richness of the available data (see for a review [23]).

To model the population dynamics of CFSE-labelled, HIV-specific CD8 T cells *ex vivo*, we consider the generation-structured (compartmental) model with time-lag, initially proposed to describe PHA-based T cell stimulation [24]. Biologically, it corresponds to the Smith-Martin view of the cell cycle kinetics [25]. Each population *N*_*i*_(*t*) representing the cells divided (*i*) times (*i* = 0,…,*I*_max_) is split into two subpopulations, the resting $N_{i}^{r}(t)$ and cycling $N_{i}^{c}(t)$ cells, as follows: $N_{i}(t)=N_{i}^{r}(t)+N_{i}^{c}(t)$. The rates of change of $N_{i}^{r}(t)$ and $N_{i}^{c}(t)$ with time are represented by the following set of delay differential equations:

$$\begin{array}{l} \frac{d}{dt}N_{0}^{r}(t)=-\left(\alpha_{0}+\beta_{0}\right)N_{0}^{r}(t),\;N_{0}^{r}(s)=0,s\in \left[-\tau_{0},0\right),N_{0}^{r}(0)=N_{0};\\ \frac{d}{dt}N_{i}^{r}(t)=-\left(\alpha_{i}+\beta_{i}\right)N_{i}^{r}(t)+2\alpha_{i-1}N_{i-1}^{r}(t-\tau_{i}),\;N_{i}^{r}(s)=0,s\in \left[-\tau_{0},0\right],i=1,\ldots ,I_{\max};\\ \frac{d}{dt}N_{i}^{c}(t)=\alpha_{i}\left(N_{i}^{r}(t)-N_{i}^{r}(t-\tau_{i})\right),\;N_{i}^{c}(s)=0,s\in \left[-\tau_{0},0\right],i=0,\ldots ,I_{\max}-1. \end{array}$$

Here *α*_*i*_ is the cycle phase transition rate of the *i*-th generation, *β*_*i*_ is the death rate of the *i*-th generation, and *τ*_*i*_ is the duration of the *i*-th division. We use the simplifying assumption that the last obtained generation does not divide. The derived system of linear delay differential equations allows an analytical solution as previously shown [24]. To estimate the per capita turnover rates of T cells with and without blockade of PD-L1, using the time series data on the generation structure of HIV Gag-stimulated T cells that constitute some small fraction *θ* of the total population of labelled cells *N*_0_, the dynamics of the non-Gag-specific T cells needs to be taken into account as well since they constitute a large fraction of labelled but non-dividing cells (see Fig 2B). Therefore, the following equation for the decay of non-specific cells was added to the above model, and the initial data were modified accordingly:

$$\frac{d}{dt}N_{0}^{non-specific}(t)=-\beta \cdot N_{0}^{non-specific}(t),\;N_{0}^{non-specific}(0)=(1-\theta )\cdot N_{0};\;N_{0}^{r}(0)=\theta \cdot N_{0}.$$

To validate the procedure for the histogram decomposition and the mathematical model of T cell growth, we compared the results of the parameter estimates for PHA-stimulated CD8 T cells from two healthy donors (CP and JA) [26], obtained using the division-structured ODE model [27], the division-structured model with time delay [24], and the Cyton-type model [26]. The summary table of the best-fit estimates for the population doubling times and the generation times are presented in S1 Table. The estimates are overall consistent.

To proceed with the parameter estimation using the HIV Gag-specific data, a systematic and rigorous assessment and ranking of the plausible data-fitting scenario is needed. Indeed, the number of data points in time series data is rather limited with respect to the number of parameters in the model, i.e. 24 measurements versus 16 parameters. Therefore, a reduction in the model parametric complexity is required to provide a most parsimonious description of the data. Note that this is a common practice in model-based CFSE analyses [24,26,28–30]. We used the information-theoretic approach in conjunction with the maximum-likelihood parameter estimation as described in our previous studies [27,30,31]. In fact, we solved 360 inverse problems (data fitting) by considering all possible combinations of various simplifying assumptions about the generation-dependent variation of cell division and death parameters:

1. Activation rates *α*_*i*_;
  - Do not depend on division number
  - The first division has a different rate than all the following ones
  - Depend on division number
2. Cell cycle durations *τ*_*i*_:
  - The first division has a different duration compared to the other ones
  - The first and second divisions have different durations than the subsequent ones
  - Depend on division number
3. Death rates *β*_*i*_:
  - All are equal to zero
  - Only the first division has a none zero death rate
  - Do not depend on the division number
  - The first division has a different rate than all the following ones
  - Depend on division number
4. Fraction of HIV Gag-specific cells *θ*
  - Constant
  - PD-L1 blockade-dependent

Let us consider the vector of the model parameters $p=\left[\alpha_{1},\ldots ,\alpha_{I_{\max}};\beta_{1},\ldots ,\beta_{I_{\max}};\tau_{1},\ldots ,\tau_{I_{\max}-1};\theta \right]$.

The best-fit estimates of the parameters p* are given by the solution of the optimization problem that minimizes the squared deviation between the time-series data $Y_{\mathrm{obs}}\equiv {\left\{t_{k},{\left\{N_{k,i}\right\}}_{i=1}^{I_{\max}}\right\}}_{k=1}^{K}$ and the model solution curve (**y**(*t*,**p**),*t*≥0): $p^{*}=\arg\;\underset{p}{\min}\Phi \left(Y_{\mathrm{obs}},y(t,p)\right)$, where Φ(**Y**_obs_,**y**(*t*,**p**)) is the mismatch function. In the case of the Gag-specific stimulation, we have two datasets for parameter estimations for each donor: without PD-L1 blockade $Y_{\mathrm{obs}}^{\mathrm{iso}}\equiv {\left\{t_{k},{\left\{N_{k,i}^{\mathrm{iso}}\right\}}_{i=1}^{I_{\max}}\right\}}_{k=1}^{K}$ and with PD-L1 blockade $Y_{\mathrm{obs}}^{\mathrm{block}}\equiv {\left\{t_{k},{\left\{N_{k,i}^{\mathrm{block}}\right\}}_{i=1}^{I_{\max}}\right\}}_{k=1}^{K}$. We assumed that the blockade affects a subset of model parameters [{*α*_*i*_, *β*_*i*_}, *τ*_*i*_, *θ*] or their combination. In this case, the best fit estimates of the cell growth parameters **p** = [**p**^invariant^,**p**^drug-affected^] can be obtained by minimizing the function $\Phi \left(Y_{\mathrm{obs}},y(t,p)\right)=\Phi \left(Y_{\mathrm{obs}}^{\mathrm{iso}},y(t,p)\right)+\Phi \left(Y_{\mathrm{obs}}^{\mathrm{block}},y(t,p)\right)$.

The comparison of the Akaike index for various combinations of simplifying assumptions (S4 Fig) suggested that the model version with activation rates depending on the division number (*α*_*i*_ ≠ *α*_*j*_,*i* ≠ *j*), all death rates equal to zero (*β*_*i*_ ≡ 0,*i* ≥ 1) and time delays equal for generations higher than the first one (*τ*_*i*_ = *τ*_*j*_, {*i*,*j*} ≥ 2), maximally reduces the value of the Akaike criteria. We also studied the Akaike criteria values, corresponding to different subsets of invariant and drug-affected parameters for the five donors. Their analyses shows that for some of the donors the smallest values of the Akaike criteria is achieved when only {*α*_*i*_, *β*_*i*_} are drug-affected, and for others–when only *θ* is drug-affected. Therefore, we formulated and tested the following assumptions (see Fig 1A):

Assumption 1. The PD-L1 blockade effect is caused by the acceleration of proliferation (reduction of doubling times) due to an enhancement of the function of exhausted T cells [5,6,12,19]. Mathematically, this implies that the kinetic parameters of cell growth are different but not the fraction of the responding cells.

Assumption 2. The PD-L1 blockade effect on T cell proliferation is due to an increase of the specific precursor number, i.e., the component of the parameter vector. From the biological point of view, this assumption means that PD-L1 blockade directly increases the number of reactive cells by reducing the amount of assessable PD-1 receptors on the T cell membrane underlying the functional impairment.

**Fig 3:**
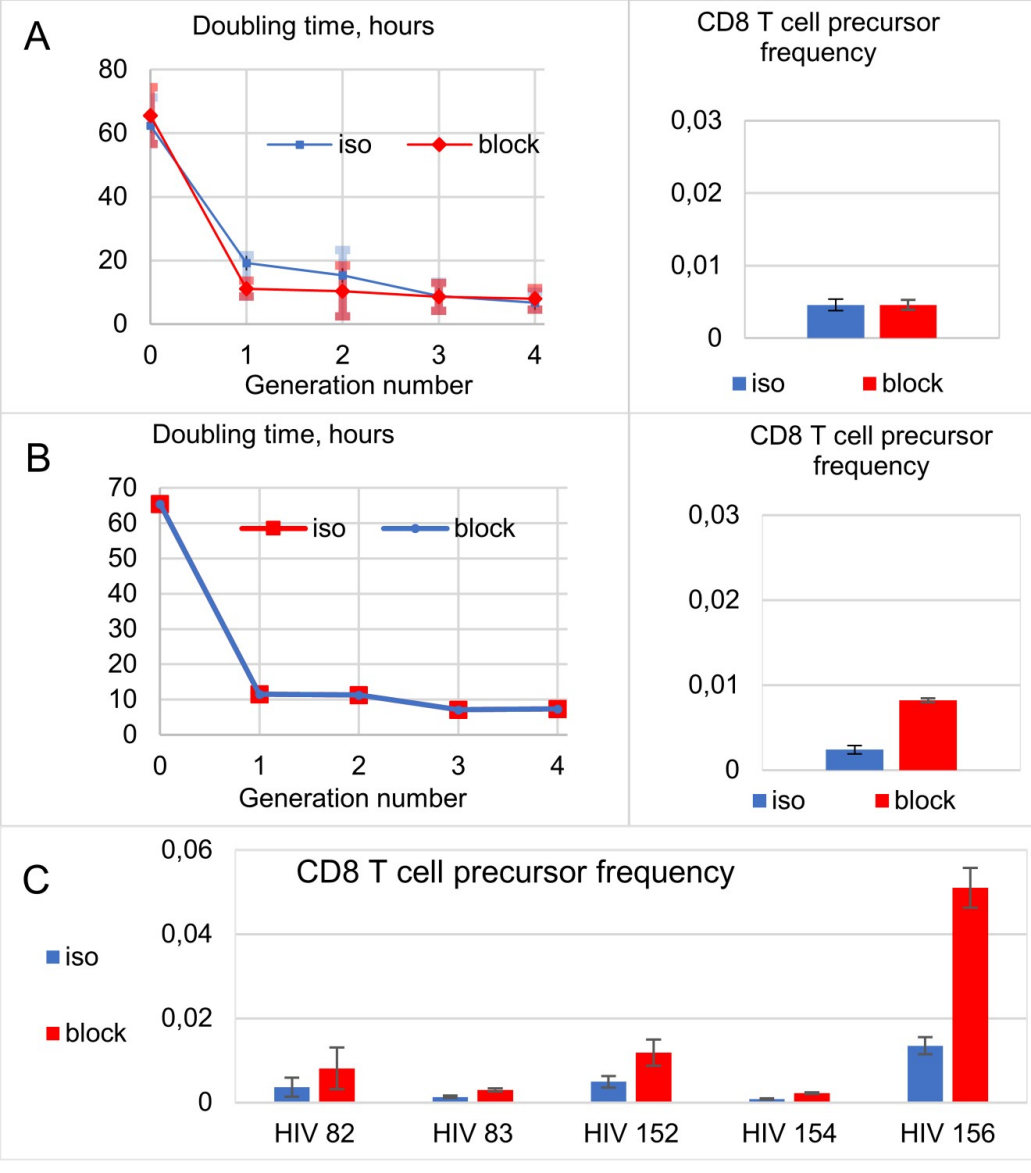
Estimated doubling times and precursor frequencies for HIV Gag-specific CD8 T cell growth after PD-L1 Blockade. Analysis of donor 82 data under the assumption of equal precursor frequencies (%), assumption 1 of Fig 1(A) or equal turnover rates, assumption 2 of Fig 1(B). The estimated increase of the CD8 T cell precursor frequency under assumption 2 for all five analysed blood donors is shown in (C). Iso, isotype control; block, PD-L1 blockade.

The estimated cell population doubling times and the precursor frequencies for HIV Gag-specific CD8 T cell growth after PD-L1 blockade are shown in Fig 3. We present the results for the donor 82 data under the assumption of equal precursor frequencies (%), assumption 1 of Fig 1 (Fig 3A), or equal turnover rates, assumption 2 of Fig 1 (Fig 3B). The estimated increase of the CD8 T cell precursor frequency under assumption 2 for all five analyzed blood donors is shown in Fig 3C.

### Linking HIV infection phenotypes with virus-host interaction parameters

#### Definition of HIV infection phenotypes

The control and progression of HIV infection is characterized by a spectrum of phenotypes for which a set of consensus definitions was proposed in 2014 [18]. The definitions categorize the heterogeneity in viral control, immune status and the disease progression, and can be further quantitatively expressed as follows:

- **P-HVL**—progressors (CD4+ T lymphocytes <200/*μl* blood in chronic phase), high viral load (>10000 copies/ml blood in chronic phase) [18,32]
- **P-MVL**—progressors, medium viral load (>2000 copies/ml blood in chronic phase)
- **P-VC**—progressors, viral controllers (<400 copies/ml blood in chronic phase)
- **SP-VC**—slow progressors (CD4+ T lymphocytes 200-500/*μl* blood in chronic phase), viral controllers
- **SP-HIC**—slow progressors, HIV controllers (HIV undetectable)
- **LTNP-HIC**—long-term nonprogressors (CD4+ T lymphocytes > 500/*μl* blood in chronic phase), HIV controllers

To quantify the PD-L1 blockade-dependent gain of CD8 T cell proliferation on viral loads and CD4 T cell counts, we used these six basic HIV phenotypes (Fig 1C) as a reference data set and took reported mean values for CD4 T lymphocyte counts and viral loads from clinical datasets [33,34]. We then formulated a mathematical model of HIV control in the steady state phase of an HIV infection and extended its components describing T cell proliferation by considering the cohorts of cells differing in the number of completed divisions (similar to the approach presented in [31]).

#### Mathematical modelling of HIV infection phenotype-specific CTL-mediated control

**Fig 4:**
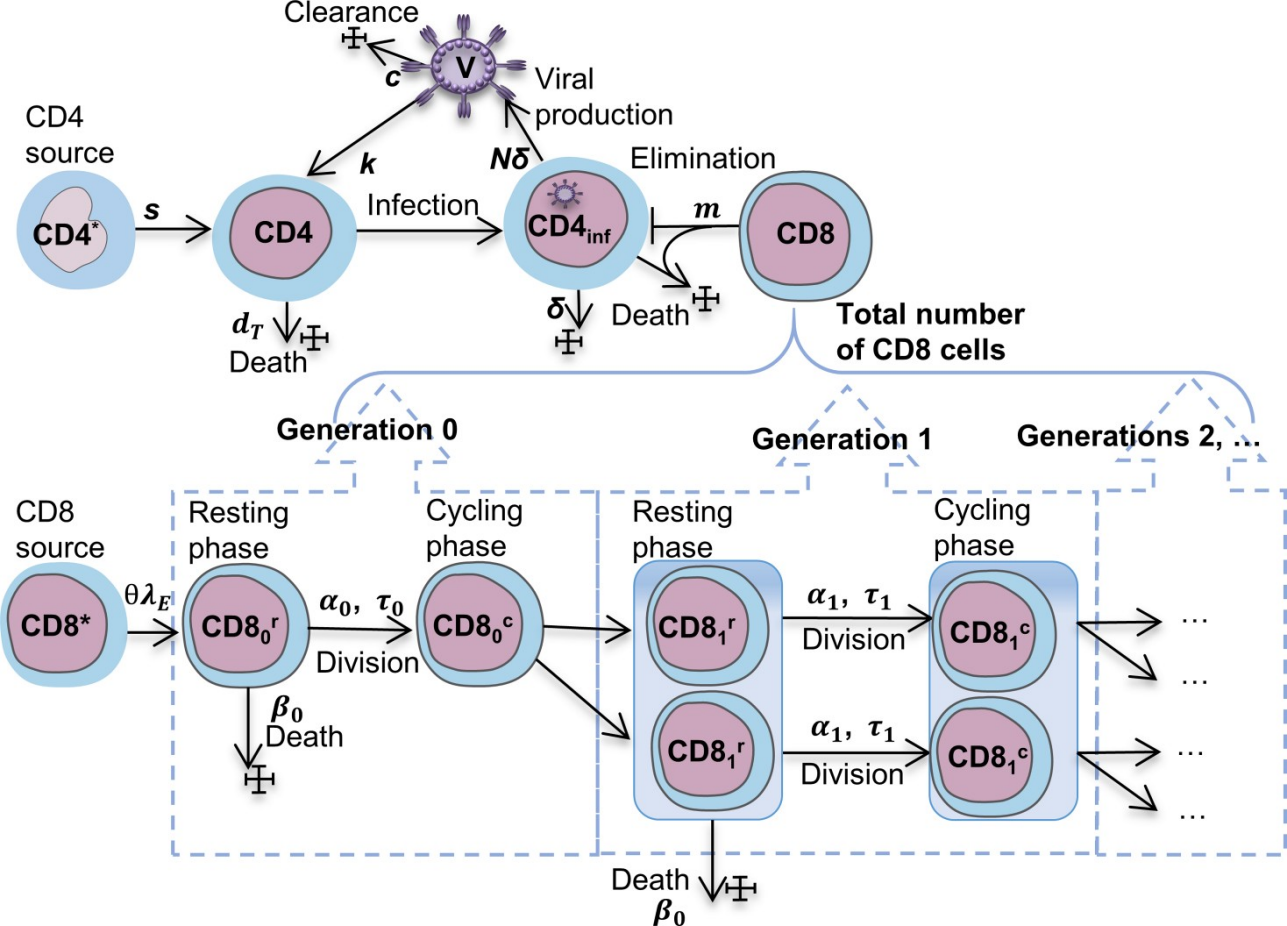
Scheme of the mathematical model of CTL-mediated control of HIV. The upper part of the Fig shows the general model scheme and set of parameters for HIV control by HIV-specific CD8 cytotoxic T cells as described [36]. The division-structured population dynamics of CTL shown below is based on the model described earlier [24].

The biological scheme of the model is shown in Fig 4. The model considers the population dynamics of uninfected CD4 T cells *(T)*, productively infected CD4 T cells *(I)*, HIV load *(V)*, HIV-specific CD8 T cells *(E*) specified according to the so-called `generalized consensus' basic model [26,35,36] and an additional set of equations describing a division-structured CTL expansion as can be derived from CFSE analysis. The equations of the generalized consensus ODE model for the *T-I-V-E* system are:

$$\frac{d}{dt}T(t)=s-d_{T}T-kV(t)T(t)$$

$$\frac{d}{dt}I(t)=kV(t)T(t)-\delta I(t)-mE(t)I(t),$$

$$\frac{d}{dt}V(t)=N\delta I(t)-cV(t)-kV(t)T(t),$$

$$\frac{d}{dt}E(t)=\lambda_{E}+\left(\frac{b_{E}I(t)}{k_{b}+I(t)}-\frac{d_{E}I(t)}{k_{d}+I(t)}\right)E(t)-\delta_{E}E(t)$$

where *E*(*t*) stands for the density of HIV-specific CTLs.

#### Model extension incorporating PD-L1 blockade-dependent CTL expansion

The mathematical model of CTL-mediated HIV control in conjunction with the estimates of the *ex vivo* gains in CTL expansion provides a tool for predicting the CTL-mediated short-term effects of PD-L1 blockade on viral load and the CD4 T cell number. For this, we extended the consensus model with the following equations using the Gag-specific parameters of the CTL response to PD-L1 blockade estimated from CFSE dilution data above:

$$\frac{d}{dt}E_{0}^{r}(t)=\theta \lambda_{E}-\delta_{E}E(t)‐(\alpha_{0}+\beta_{0})E_{0}^{r}(t),\;E_{i}^{r}(s)=0,\;s\in [-\tau_{i},0),E_{i}^{r}(0)=\theta E_{0};$$

$$\frac{d}{dt}E_{i}^{r}(t)=-(\alpha_{i}+\beta_{i})E_{i}^{r}(t)+2\alpha_{i-1}E_{i-1}^{r}(t-\tau_{i-1}),\;E_{i-1}^{r}(s)=0,\;s\in [-\tau_{i},0],\;i=1,\ldots ,I_{max}$$

$$\frac{d}{dt}E_{i}^{c}(t)=\alpha_{i}(E_{i}^{r}(t)-E_{i}^{r}(t-\tau_{i})),\;E_{i}^{c}(s)=0,\;s\in [-\tau_{i},0],\;i=0,\ldots ,I_{max}$$

with *I*_max_ = 5.

Under this setting, HIV-specific CTLs are a heterogeneous population composed of cells differing in the number of completed divisions $E(t)=\sum_{i}^{I_{\max}}\left(E_{i}^{r}(t)+w_{i}E_{i}^{c}(t)\right)$. The parameter ${\left\{w_{i}\right\}}_{i=1}^{I_{\max}}$ is introduced to take into account that the different generations of CTL can differ in their killing capabilities. Indeed, antigen-induced CD8 T cell proliferation resulted in the progressive differentiation towards an effector cell status with a division-correlating effector mRNA expression and increase of ex vivo cytotoxicity [37]. Moreover, a more recent study provided data on the dynamics of the killing capacity of CTL showing that most CTLs initially behave as low-rate killers, while some of them develop as high-rate killers at later time points [38]. In our present analysis, we considered that *w*_*i*_ = 1.

**Fig 5:**
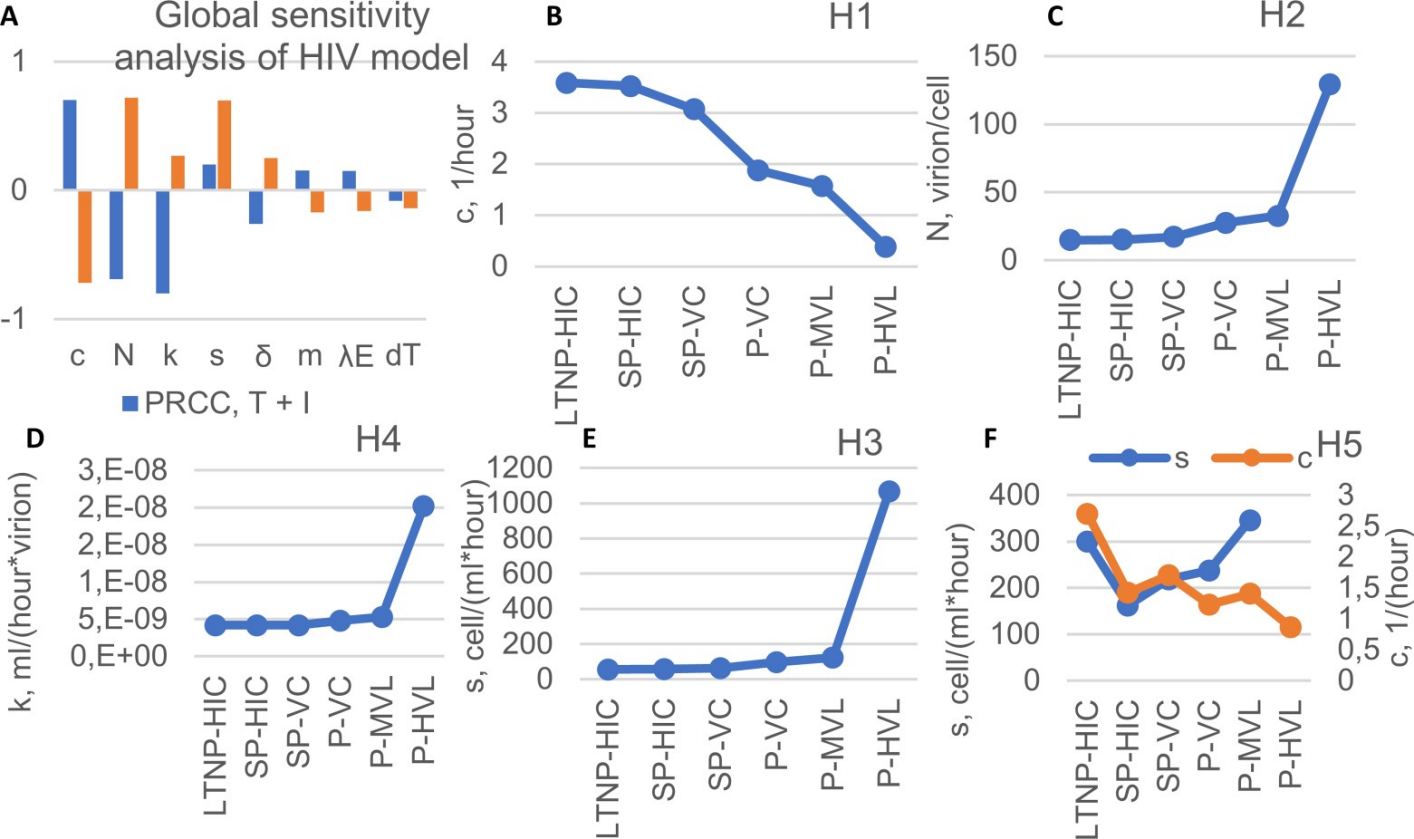
Global sensitivity analysis and prediction of model parameter values for the different HIV infection phenotypes. (A) Partial rank correlation coefficients (PRCC) for the total number of CD4 T cells (T+ I) and viral load (V) in an HIV infection. Parameters are sorted in descending order according to the PRCC value. Parameters with higher PRCC values (c, N, s, k) make a greater contribution to the change of viral load and CD4 T cells. (B-F) Parameter values were estimated for different HIV infection phenotypes according to hypotheses 1 to 5 (H1 to H5).

The extended model (see the state variables in S2 Table) with parameters {*α*_*i*_,*β*_*i*_,*τ*_*i*_,*θ*} corresponding to the Gag-specific CTL response of donor 156 before PD-L1 blockade was used to estimate the parameters underlying the six basic phenotypes presented in Fig 1C (see also S3 Fig). To proceed with the parameter estimation, we performed the global sensitivity analysis using the LHS-PRCC technique based on Latin hypercube sampling and partial rank correlation coefficient [39]. This provided a basis for ranking the model parameters according to their impact on the steady states of viral load and CD4 Т-lymphocytes as shown in Fig 5A. According to Muller et al. [40], small variations in multiple parameters account for wide variations in HIV-1 set-points. Based on these observations, we considered those parameters as HIV phenotype controlling that displayed higher global sensitivity coefficients. Because the phenotype-specific data refer to steady state values of viral load and CD4 T cell count, the number of parameters that can be estimated and further used has to be limited to one or two. The results of the computational solution of the resulting set of phenotype data-fitting problems allowed us to specify the following set of five hypotheses (H1 to H5) for the parameterization of the observed phenotypes: H1 –{c}, H2 –{N}, H3 –{s}, H4 –{k}, and H5 –{s,c} shown in Fig 5B to 5F, respectively (see S3 Table for parameter definitions). The loss of HIV infection control observed from LTNP-HIC to P-HVL phenotypes can be associated with the decrease of virus elimination rate (H1), an increase in the virus production rate (H2), an increase in the supply of uninfected target cells (H3), an increase in the infection rate (H4), or a reduction of the virus elimination rate with concurrent increase of target cell production (H5). The consequences of the PD-L1 blockade were examined for the above five hypotheses for the chronic infection phenotypes and the two assumptions for the HIV-specific T cell responsiveness (doubling time, precursor frequency).

### HIV infection phenotype- and patient-specific predictions of clinical benefit of PD-L1 blockade therapy

#### Consideration of a PD-L1 blockade-dependent cytotoxic CD8 T cell gain

**Fig 6:**
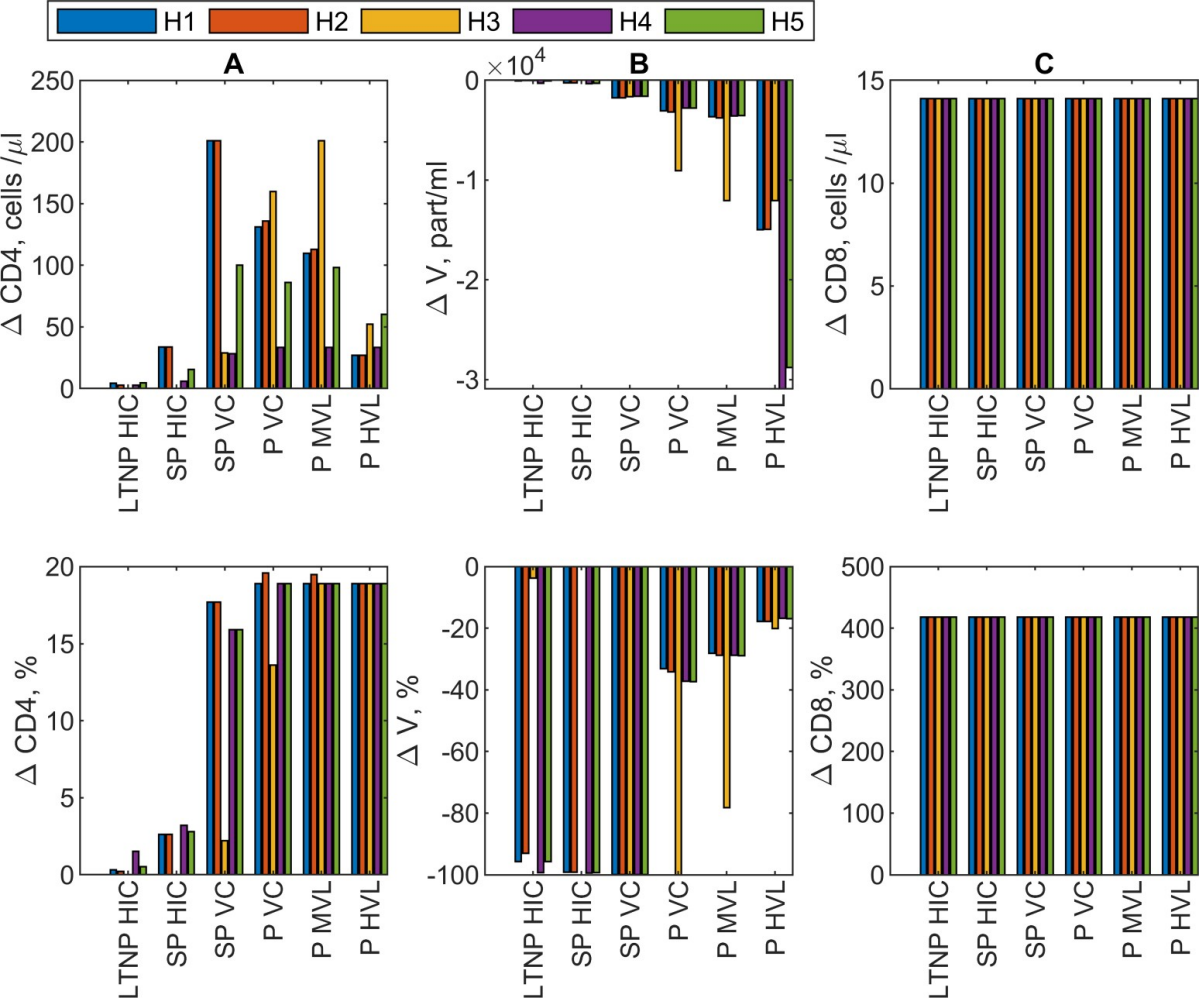
Prediction of the effect of PD-L1 blockade on total CD4 T cells, virus load and HIV-specific CD8 T cells for different HIV infection phenotypes under hypotheses 1 to 5. CD8 T-lymphocyte proliferation parameters from donor HIV-156 are used as illustrative example. The predicted change in the number of CD4 T cells (A), virus load (B) and HIV-specific CD8 T cells (C) are given in an absolute (upper) or relative (lower) scale. All predictions are based on assumption 1. HIV infection phenotypes are as in Fig 1. The color code for H1 to H5 is given.

For each phenotype pattern, i.e. for H1 to H5, we compared the model solutions using the CTL response parameters before and after PD-L1 blockade for all HIV patients. The CD8 T-lymphocyte proliferation parameters from donor HIV-156 are used as illustrative example. The results are summarized in Fig 6. The predicted change in the number of CD4 T cells (A), virus load (B) and HIV-specific CD8 T cells (C) are given in an absolute (upper) or relative (lower) scale. All predictions are based on assumption 1. HIV infection phenotypes are as in Fig 1.

The model predicts that the viral load should decrease while the number of CD4 T-lymphocytes increase after PD-L1 blockade. As far as the CD4 T cell gain is concerned, both absolute and relative changes appear to be stronger for poorer HIV controllers. The absolute changes in the virus load are stronger for poorer controllers but the relative changes show an opposite relationship.

**Fig 7:**
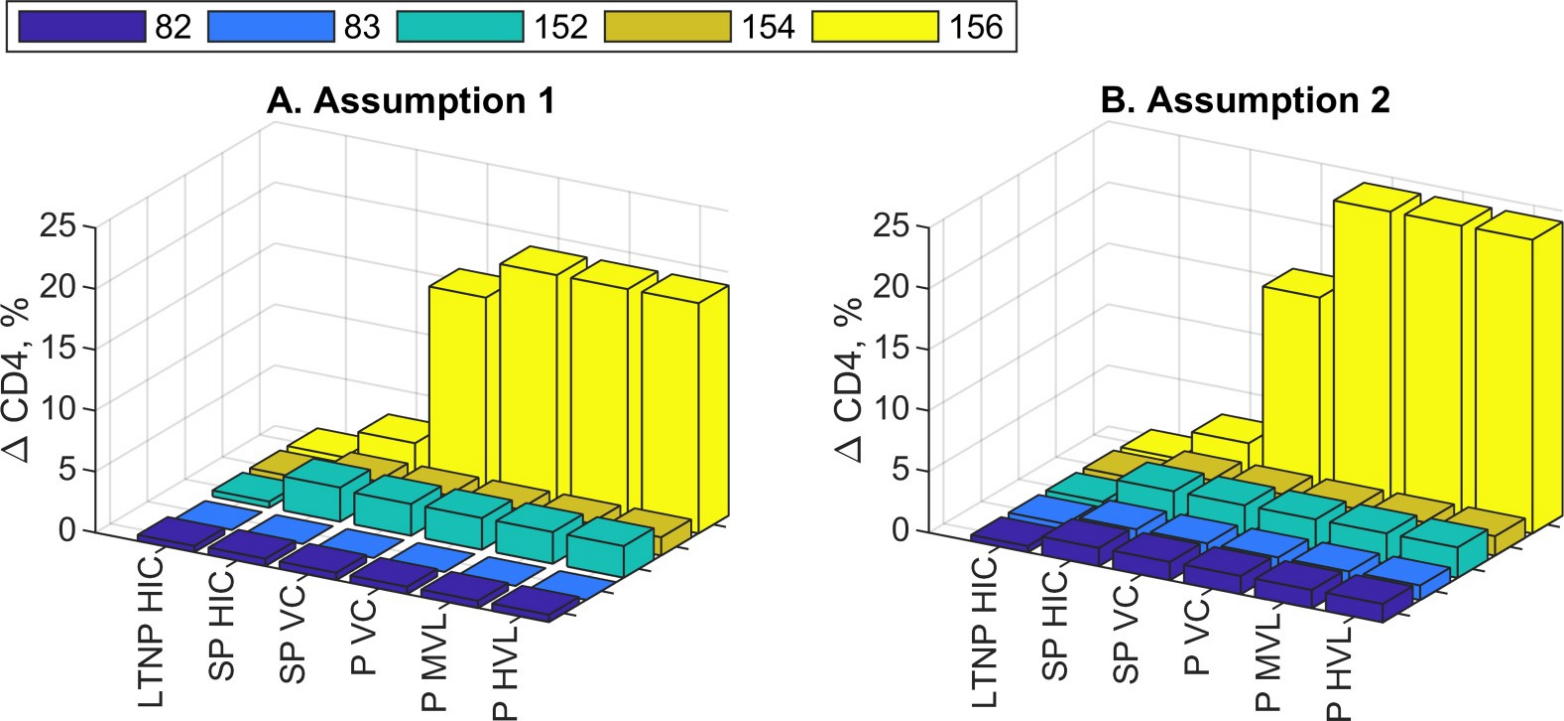
Donor-specific prediction of relative CD4 T cell increases for different HIV infection phenotypes. Parameters of the CD8 T cell proliferative responsiveness of all 5 blood donors were estimated from the CFSE profiles after PD-L1 blockade according to assumptions 1 (A) or 2 (B) under hypothesis 5. HIV infection phenotypes are as in Fig 1.

Donor-specific predictions of the relative CD4 T cell increases for different HIV infection phenotypes are shown in Fig 7. The Parameters of the CD8 T cell proliferative responsiveness of all 5 blood donors were estimated from the CFSE profiles after PD-L1 blockade according to assumptions 1 (A) or 2 (B) under hypothesis 5 and for HIV infection phenotypes as in Fig 1. All the donors show different responses. The strongest rise of the CD4 T cells is observed in donor 156 who also has the highest increase of the CD8 T cell (see Fig 3C). Importantly, the characteristics of the CD4 T cell responses are robust with respect to both assumptions.

Up to here, we have only considered PD-L1 blockade effects on cytotoxic CD8 T cells. However, PD-1 is a marker of lymphocyte activation and expressed on several lymphocyte subsets including CD4 T cells and B cells. As a consequence, anti-PD-L1 treatment can have multiple effects beyond those on CD8 T cells. To expand our analyses accordingly, we next incorporated data from the HIV-specific CD4 T cell expansion of the 5 patients tested.

#### Consideration of a PD-L1 blockade-dependent CD4 T cell increase

Activated CD4 T cells are the prime target cells of HIV. Activation of CD4 T cells entails the expression of PD-1 that, when interacting with PD-L1, can lead to CD4 T cell exhaustion. Consequently, the application of a PD-L1 blockade can result in an increase of activated T cells and thus more target cells for HIV expansion. This is considered as one of the causes of the observed initial rise of the plasma viremia in SIV-infected monkeys following PD-1 blockade in late-phase-treated and some of early-phase-treated animals [9].

**Fig 8:**
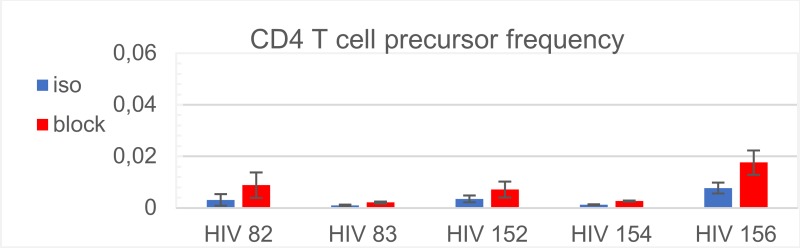
Estimated precursor frequencies [*θ*] for the HIV Gag-specific CD4 T cell rise after PD-L1 blockade. The estimated fraction of the CD4 T cells from the total CD4 T cell number under assumption 2 that PD-L1 blockade increases the number of specific precursor cells for all five analysed blood donors is shown. Iso, isotype control; block, PD-L1 blockade.

To evaluate the effect of PD-L1 blockade on HIV target cell expansion, we first analysed our original data on CFSE dilution of Gag-specific CD4 T cell proliferation (S1B Fig). Experimental conditions and histogram shapes are similar to those of patients’ CD8 T cell analyses. Since assuming, as underlying mechanism of a PD-L1 blockade, either a kinetic parameter change of cell growth or an increase in precursor number [*θ*] gave similar outcome predictions, we followed the simpler second assumption, i.e. that it is the number of responding CD4 T cells that is affected by anti PD-L1. The results of the estimation of [*θ*] for five patients are presented in Fig 8.

To then incorporate the observed PD-1 blockade-mediated CD4 T cell increase into the T-I-V-E model of CTL-mediated HIV control, we modified the equation for the rate of change of the uninfected CD4 T cell population and the CD4 T cell number as follows:

$$\frac{d}{dt}T(t)=s-d_{T}T-kV(t)T(t),\;T(0)=T_{0}\;‐‐before\;PD‐L1\;blockade,$$

$$\frac{d}{dt}T(t)=((1-\mu )+\mu \frac{\theta_{block}}{\theta_{iso}})s-d_{T}T-kV(t)T(t),\;T(0)=((1-\mu )+\mu \frac{\theta_{block}}{\theta_{iso}})T_{0}\;\mathrm{‐‐}after\;PD‐L1\;blockade.$$

**Fig 9:**
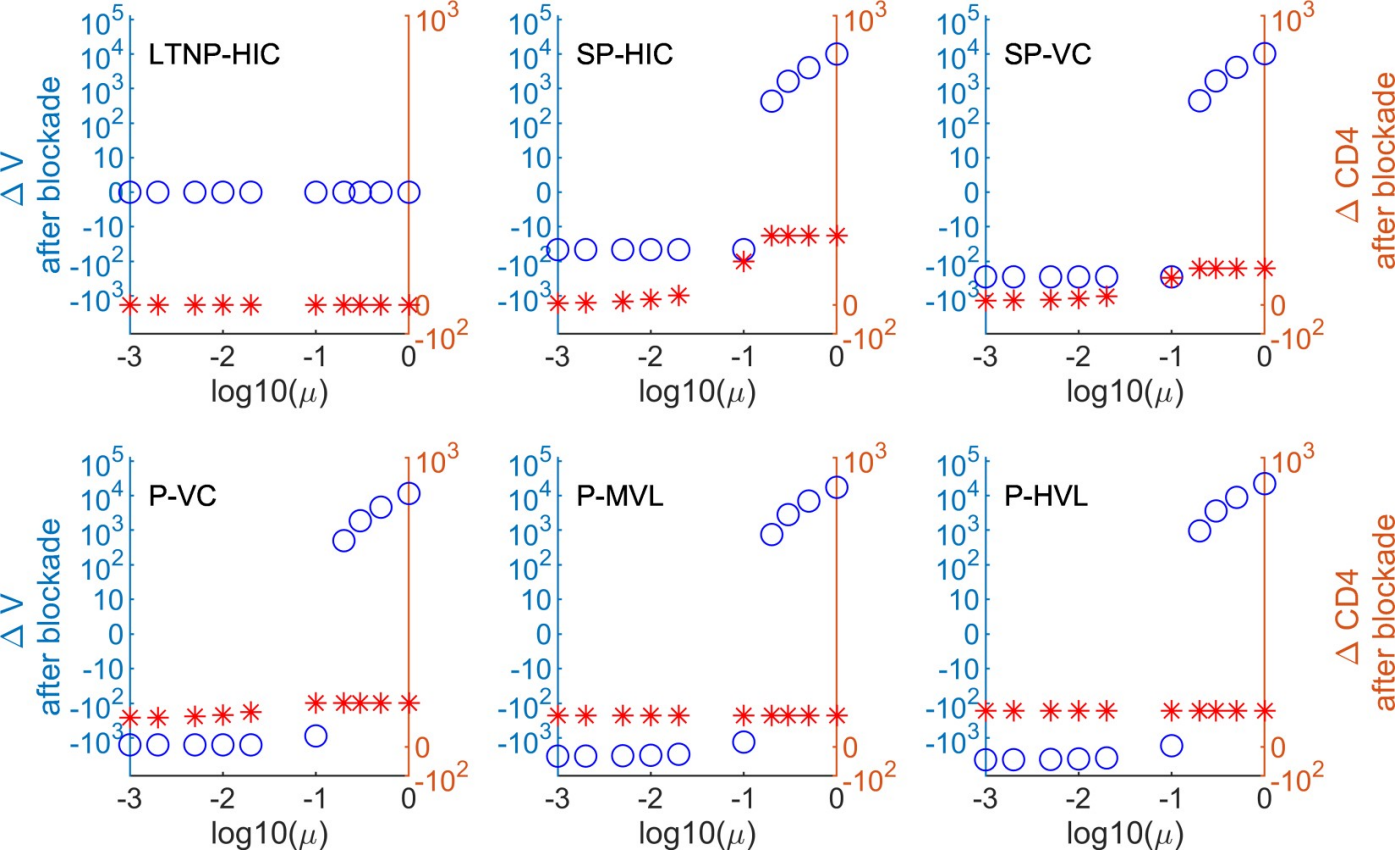
HIV infection phenotype-specific predictions of PD-L1 blockade-mediated changes of virus load and CD4 T cell counts considering gains of HIV-specific CTL and HIV-infectible CD4 T cell targets. Predictions based on the determined increases of HIV Gag-specific CD8 and CD4 T cells of infected donor 156 are shown (other donors are represented in S7 Fig). Δ*V* (open circles) refers to an absolute change in viral load. Δ*CD*4 (asterisks) indicates an increase in CD4 T cell numbers. *μ*, the fraction of restored activated CD4 T cells after PD-L1 blockade.

Here *μ****ϵ***[**0**,**1**] is the fraction of restored activated CD4 T cells that contribute to the target cell population. The ex vivo patients’ data suggest that the fraction of Gag-specific CD4 T cells ranges from 0.002 to 0.05 (Fig 8). However, the frequency of PD-1-expressing CD4 T cells that can potentially respond to therapy is much larger and may vary from about 10% to up to 100% in the memory T cell subset [41,42]. To cover the whole range of potentially responding CD4 T cells that may be activated and become target cells for HIV replication, we incorporated 0.001 ≤ *μ* ≤ 1. The resulting quantitative predictions in terms of viral load changes and increase of CD4 T cells for each donor and HIV infection phenotypes are summarized in Fig 9 and S7 Fig. The model predicts that the viral load is reduced after PD-L1 blockade only when the fraction of activated CD4 T-lymphocytes which recover (μ) is below a certain threshold. This threshold is about 1% for donors 82 and 83, 2% for donors 152 and 154, and 10% for donor 156. Of note, donor 156, in contrast to the others, is characterized by a substantial restoration of the CD4+ T cell population after PD-L1 blockade. The HIV infection phenotypes with lesser virus control are characterized by a larger reduction of the viral load. There is no visible effect for long-term non-progressors (LTNP-HIC). The overall dependence of the viral load reduction on (μ) results in a switch-like characteristics between a reduction-mode and an increase-mode. The beneficial effect in terms of the switch threshold is associated with a more significant increase in the frequency of virus-specific CD8 T cells., e.g. donor 156 versus donor 83 (see also Fig 3C).

#### Consideration of a PD-L1 blockade-dependent neutralizing antibody increase

Antibody-producing B cells are also prone to PD-1-mediated exhaustion [43]. Therefore, PD-L1 blockade could increase neutralizing antibody production. Indeed, reactivating exhausted B cells in SIV-infected monkeys by an anti-PD-1 treatment resulted in a 2–8 fold increase of SIV-specific binding antibody titres, and about a 2 fold increase of neutralizing antibody titres [9]. Respective data of treated HIV patients are not yet available. In our mathematical model, it is the viral clearance rate that will be strongly influenced by the presence or absence of neutralizing antibodies. Thus, to evaluate how a potential PD-L1 blockade-dependent neutralizing antibody increase would affect HIV patients’ treatment outcome, we increased the elimination rate of the virus population {c}. Due to the lack of quantitative data in HIV infection, we considered the SIV studies in monkeys [9] and assumed that a PD-L1 blockade may result in a two-fold increase of the virus clearance rate {c}. The equation for the virus population then reads:

$$\frac{d}{dt}V(t)=N\delta I(t)-2cV(t)-kV(t)T(t).$$

**Fig 10:**
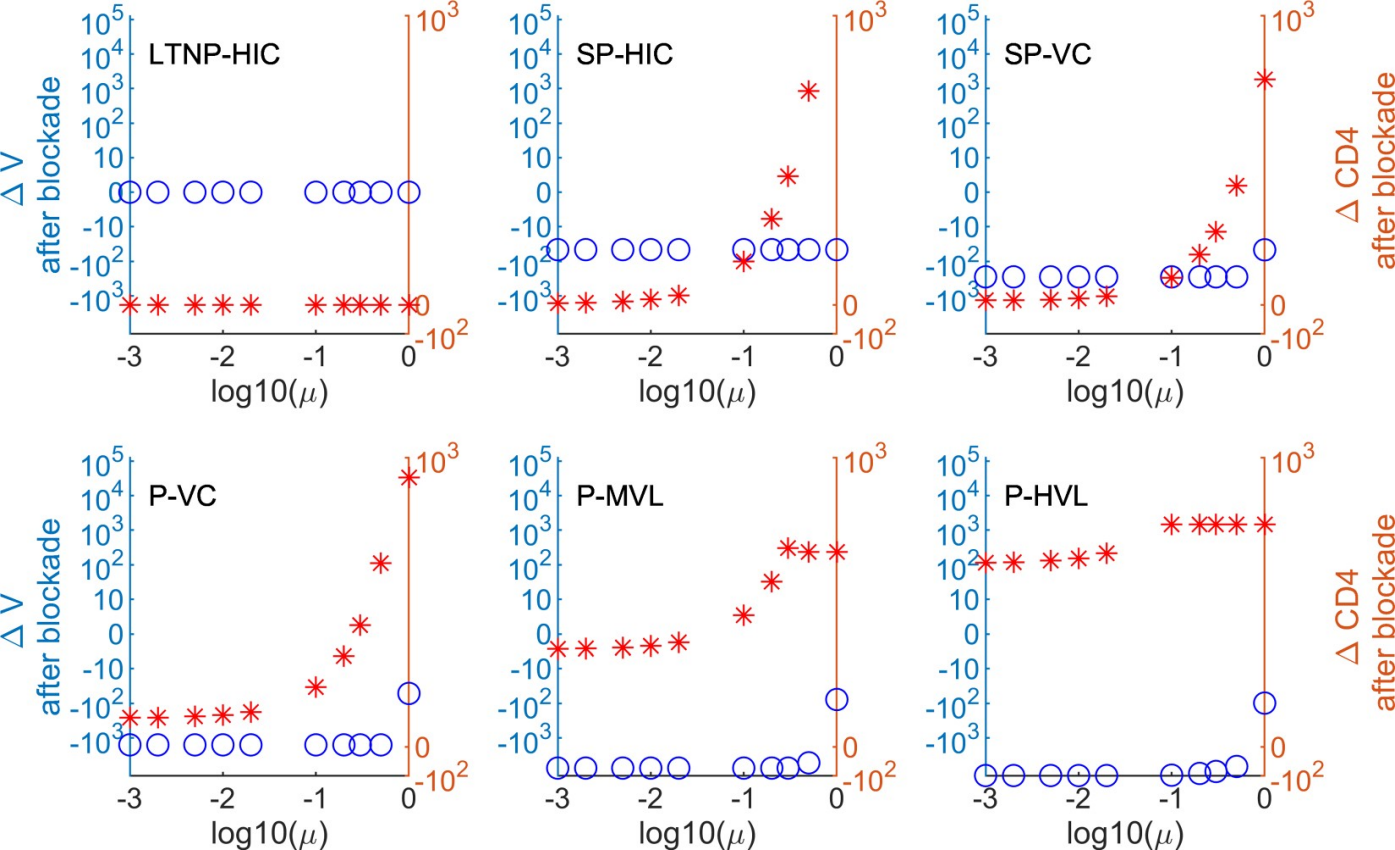
HIV infection phenotype-specific predictions of PD-L1 blockade-mediated changes of virus load and CD4 T cell counts considering gains of HIV-specific CTL, HIV-infectible CD4 T cell targets and neutralizing antibodies. Predictions based on the determined increases of HIV Gag-specific CD8 and CD4 T cells of infected donor 156 with an assumed 2-fold increase of neutralizing antibody titres are shown (predictions for the other donors are in S8 Fig). Δ*V* (open circles) refers to an absolute change in viral load. Δ*CD*4 (asterisks) indicates an increase in CD4 T cell numbers. *μ*, the fraction of restored activated CD4 T cells after PD-L1 blockade.

The modelling results after incorporating the determined PD-L1 blockade effects on CD8 and CD4 T cells, and the estimated neutralising antibody increase are shown in Fig 10 and S8 Fig.

A 2-fold increase in the elimination rate of the virus population has a dramatic impact on the response of the system as a whole. It makes a PD-L1 blockade clinically beneficial for almost the whole range of responding CD4 T cell fractions *μ*. The threshold for the net beneficial effect moves up to μ ~ 0.5 for donors 82 and 152. For the other donors, it shifts to 1. Interestingly, the less controlled HIV infection phenotypes are again characterized by a larger reduction of the viral load. There is no visible effect for long-term non-progressors (LTNP-HIC). The net impact of the PD-1 blockade on the restoration of the CD4 T cell population is remarkable. The cell population size starts to increase for μ around 0.1.

## Discussion

With this study we sought to quantify the PD-L1 blockade-mediated functional gain of the proliferative responsiveness of HIV-specific T lymphocytes and predict the potential therapeutic effect for HIV patients of different disease progression phenotypes. We found, first, that dynamic cell parameters and specific precursor frequencies could be readily estimated from time-resolved CFSE profiles despite the low frequency of HIV-specific memory CD8 and CD4 T cells within the overall PBMC population [44]. Second, with the proliferation parameter estimates as input, a CTL-based model of HIV control was generated that enabled to predict the ranges of response parameter for which patients could expect clinical treatment benefits. Together this work outlines a complete strategy imbedding ex vivo proliferation data acquisition, data analysis and mathematical model predictions for personalized immunotherapies.

Checkpoint inhibitor therapies including PD-L1 blockade have produced long-term remission of a few advanced tumors and even some cures, however, on average, only about 10–30% of treated cancer patients have a clear benefit [8]. Comparable data for treatment of HIV patients are not yet available since HIV infection was mostly an exclusion criteria in the clinical trials. Nonetheless, published case reports provided evidence for an increase of HIV-specific T cell functionality and reduction in viral reservoirs in some of the few patients studied so far [11]. These results are encouraging and biomarker that could predict clinical improvements for treated HIV patients would be of great interest for clinical decision making. The gain of function of HIV-specific CD8 T cells via PD-L1 blockade may represent such a biomarker for HIV-infected individuals as this immune cell population is directly linked with HIV control [12]. Nonetheless, checkpoint inhibitor therapies also increase activated CD4 T cell numbers and thus the targets for HIV expansion, and may increase neutralizing antibody titres by revitalization of exhausted B cells. This additional interplay between HIV-expanding and HIV-restricting processes clearly adds complexity to the overall biology of these treatments. However, it may be less important in vivo since immunotherapies are now recommended only when HIV levels are well controlled by combined antiretroviral drugs.

PD-L1 blockade increased the HIV-1 Gag-specific responsiveness of CD8 and CD4 T cells from all 5 examined HIV blood donors albeit to different extent (see CFSE histograms on S1, S2 and S5 Figs). The frequency of antigen-specific cells within the PBMC population is low and thus, the CFSE histograms are too noisy to apply complex proliferation analyses [26]. However, with a simple mathematical model of cell proliferation with time delays [24] that we first validated using polyclonally stimulated CD8 T cells from healthy blood donors, we derived proliferation parameters that showed a good approximation of our experimental data (S6 Fig). A priori, the gain of antigenic responsiveness of HIV-specific T cells caused by PD-L1 blockade may be due to changes in the dynamic parameters of cell proliferation or to an increased number of responsive cells thus, an increased precursor frequency. Both assumptions were tested in our analyses and led to similar CFSE histogram descriptions (S1 and S5 Figs) and the same prediction of treatment benefits for a subgroup of HIV phenotypes (Fig 7). Thus, our predictions are robust with respect to the mechanism that may cause the antigenic responsiveness increase.

Antigen-specific cytotoxic CD8 T cells are a major component in the control of an HIV infection [17,45–47] and cancers [48]. We here developed and calibrated a CTL-based model of HIV infection control (short-time response) to predict the response of HIV-infected patients to PD-L1 blockade. The model-driven analyses of CFSE data on proliferative performance of virus-specific CTLs in conjunction with several hypotheses about the control processes of different HIV infection phenotypes allowed us to assess the spectrum of reactions to PD-L1 interventions and to propose biomarker for therapy success. These latter include the gains of CTL responsiveness and neutralizing antibody titres, and the fraction of responding CD4 T cells. Together, the developed systematic analytical approach to quantifying the immune therapy effect in a patient-specific manner provides a mechanistic tool for treatment evaluation.

Our cytotoxic T cell model of HIV control can be easily adapted to describe the control of tumor growth. However, proliferation parameter estimates of tumor-specific T cells cannot be as easily determined as for HIV because tumor-specific T cell epitopes are less well defined, vary between tumor types, and are often observed with lower frequencies. Thus, the adaptation of our global approach to predict therapy outcomes in cancer requires modifications and assay improvements at several levels.

In conclusion, we here present a modelling approach that incorporates time-resolved CFSE proliferation analyses of antigen-specific CD8 and CD4 T cells into a population model of HIV infection dynamics. It allows to use ex-vivo-determined PD-L1 blockade effects from individual HIV blood donors and predict the in vivo outcome. While our approach is still simplistic and thus covers not all aspects of an HIV infection, it provides a general framework of how to link experimental biomarker studies with patient outcome predictions. The more we will be able to quantitate and formalize mechanistic relationships in complex diseases, the closer we will be to personalize treatment strategies.

## Materials and methods

### Donors and datasets

Data for PHA stimulation was collected from two healthy donors (CP and JA). Data for HIV Gag-specific stimulation was collected from five HIV infected donors that differ in viral load and CD4 T cell counts: patient 82 (CD4 = 362 cells/μl, HIV RNA = 886 copies/ml, no treatment), patient 83 (CD4 = 289 cells/μl, HIV RNA undetectable, on HAART), patient 152 (CD4>600 cells/μl, HIV RNA undetectable, on HAART), patient 154 (CD4>600 cells/μl, HIV RNA undetectable, on HAART), patient 156 (CD4>600 cells/μl, HIV RNA undetectable, on HAART). Measurement times after stimulation differ for these patients’ datasets (every 24 hours for healthy donors and HIV-infected patients 82 and 83; and at 24, 84, 96, 108, 120 and 132 hours for HIV-infected patients 152, 154 and 156).

### Proliferation assay

Peripheral blood mononuclear cells (PBMC) were isolated from blood by Ficoll density centrifugation (Invitrogen). Cells were stained with Carboxyfluorescein succinimidyl ester (CFSE) (Invitrogen), cultured with anti-CD28 and anti-CD49d antibodies (1μg/mL), and further incubated with PHA or with 1μg/mL Gag pool of overlapping peptides (Gag peptides; NIH reagent program catalogue number 8117 and 8118). Cells were harvested at the indicated time points, stained with fluorochrome-labeled anti-CD3, anti-CD4 and anti-CD8 antibodies, and analyzed by flow cytometry.

### Model verification

To show the consistency of the parameter estimation results obtained by our modelling approach with the results of previously published studies on division number-dependent proliferation rates, we considered the generation times. The generation time *Ti* is considered to be the average time between the birth and consecutive division of a cell from *i-th* generation. The formulas which can be used to estimate the generation time depend on the specific way used to model the cell division. If we use the following notation, i.e. *gi*—exponential growth rate, τ_i_—division delay, α_i_—activation rate, g(s)−generation time distribution function, then the following relationships provide the estimates of the generation times [30,49]:

$$Ti=\frac{1}{g_{i}};\;Ti=\tau_{i}+\frac{1}{\alpha_{i}};\;Ti=\int_{0}^{\infty }sg(s)ds$$

The estimated generation times for PHA-stimulated CD8 T lymphocytes from healthy donors CP and JA obtained using our model, the division-structured ODE model [27], the division-structured model with time delay [24], and the Cyton-type model [26,29], are presented in S1 Table. The obtained estimates for CD8 T-lymphocytes from healthy donors are in agreement with the values, obtained in other studies.

### Ethics statement

Ethical committee approval and written informed consent from all subjects, in accordance with the Declaration of Helsinki, were obtained prior to study initiation. The study was approved by the institution’ ethical committee CEIC- Parc de Salut Mar, Barcelona, Spain (Protocol approval number: 2013/5422/I).

## Supporting information

S1 Fig Experimental histograms and the best-fit model solutions. Blue- and red-colored areas correspond to the CFSE histograms without- PD-L1 blockade and with PD-L1 blockade, respectively. Blue line represents the division-structured CTL proliferation model solution calibrated using the CFSE dilution data without PD-L1 blockade, and red line—with PD-L1 blockade. The data-fitting problem was solved under the Assumption 1 for CD8 T cells (A) and assumption 2 for CD4 T cells (B). The model solution histograms were produced using the values of the generation-specific gaussian means and standard deviations obtained at the CFSE histogram approximation-decomposition stage. The gaussian weighting coefficients correspond to the number of cells in each generation. The first six divisions are considered.
(TIF)

S2 Fig Experimental histograms and model solution for donors 82 (A) and 83 (B). The eight first divisions are considered. Blue colored areas correspond to the histogram without PD-L1 blockade, and red areas–with PD-L1 blockade. Blue lines correspond to best-fit solutions of the division-structured CTL proliferation model without PD-L1 blockade, and red line—with PD-L1 blockade. The model parameters were estimated under Assumption 1.
(TIF)

S3 Fig Effect of PD-L1 blockade on virus and CD4 T cell values for different HIV infection phenotypes. The solid and dashed lines correspond to the model solutions without- and with PD -L1 blockade, respectively. The model solutions were obtained under Hypothesis 5. Here, ΔT is the change of the number of CD4 T-lymphocytes after PD-L1 blockade, ΔV is the change of the viral load, ΔE spec is the change of the number of the specific CD8 T-lymphocytes. The “+” symbols correspond to the initial dataset for each HIV infection phenotype, and the dots to the steady state values, both used for the model parameter estimations.
(TIF)

S4 Fig Estimates of the Akaike criterion value for various combinations of simplifying assumptions for the CFSE-labelled cell proliferation model. Each plot corresponds to a different setting for invariant and drug-affected parameter subsets, specified at the top of each figure. Each set of coloured points corresponds to one of the donors 82, 83, 152, 154, 156. Each individual point corresponds to the Akaike criterion value (y-axis) for one combination of simplifying assumptions about the generation-dependent variation of cell division and death parameters (x-axis). Blue circles correspond to minimal AIC for each donor and each combination, big black circles–to the global AIC minima for each donor. The smallest values correspond to the following combinations:
*p*^*invariant*^ = [*τ*_*i*_, *θ*], *p*^*drug−affected*^ = [{*α*_*i*_, *β*_*i*_}], *α*_*i*_ depends on division number, *β*_*i*_ = 0 for all generations, the first division has a different duration compared to the later ones (for two donors);*p*^*invariant*^ = [{*α*_*i*_, *β*_*i*_}], *p*^*drug*−*affected*^ = [*τ*_*i*_, *θ*], *α*_*i*_ depends on division number, *β*_*i*_ = 0 for all generations, the first division has a different duration compared to the later ones (for one donor);*p*^*invariant*^ = [*τ*_*i*_], *p*^*drug−affected*^ = [{*α*_*i*_, *β*_*i*_}, *θ*], *α*_*i*_ depends on division number, *β*_*i*_ = 0 for all generations, the first and second divisions have different duration compared to the later ones (for one donor);*p*^*invariant*^ = [{*α*_*i*_, *β*_*i*_}, *τ*_*i*_], *p*^*drug−affected*^ = [*θ*],*α*_*i*_ depends on division number, *β*_*i*_ = 0 for all generations, the first division has a different duration compared to the later ones (for one donor).
(TIF)

S5 Fig Experimental histograms and the best-fit model solutions for varying number of precursors. Blue- and red-coloured areas correspond to the histograms with- and without PD-L1 blockade, respectively. The blue line represents the solution of the division-structured CTL proliferation model without PD-L1 blockade, and red line with PD-L1 blockade. The data-fitting problem was solved under the Assumption 2. The model-based solution histograms were produced using the gaussian mean and standard deviation values obtained at the CFSE histograms approximation-decomposition stage. The gaussian weighting coefficients correspond to the number of cells in each generation. The first six divisions are considered.
(TIF)

S6 Fig Cell numbers, estimated from experimental histograms (points) and the best-fit model solution (solid lines) for PHA-stimulated CD8 T-lymphocytes from healthy donors CP (A) and JA (B). Each plot represents the cell population dynamics for generations from 1 (leftmost) to 5 (rightmost).
(TIF)

S7 Fig HIV infection phenotype-specific predictions of PD-L1 blockade-mediated changes of virus load and CD4 T cell counts considering gains of HIV-specific CTL and HIV-infectible CD4 T cell targets. Predictions based on the determined increases of HIV Gag-specific CD8 and CD4 T cells of infected donors 82, 83, 152 and 154 are shown. Δ*V* (open circles) refers to an absolute change in viral load. Δ*CD*4 (asterisks) indicates an increase in CD4+ T cell numbers. *μ*, the fraction of restored activated CD4 T cells after PD-L1 blockade.
(TIF)

S8 Fig HIV infection phenotype-specific predictions of PD-L1 blockade-mediated changes of virus load and CD4 T cell counts considering gains of HIV-specific CTL, HIV-infectible CD4 T cell targets and neutralizing antibodies. Predictions based on the determined increases of HIV Gag-specific CD8 and CD4 T cells of infected donors 82, 83, 152 and 154 with an assumed 2-fold increase of neutralizing antibody titres are shown. Δ*V* (open circles) refers to an absolute change in viral load. Δ*CD*4 (asterisks) indicates an increase in CD4 T cell numbers. *μ*, the fraction of restored activated CD4 T cells after PD-L1 blockade.
(TIF)

S1 Table Comparison of the estimated generation times for various models of cell proliferation. (DOCX)

S2 Table Time-dependent state variables of the mathematical model of CTL-mediated control of chronic HIV infection. (DOCX)

S3 Table Parameters of the mathematical model of CTL-mediated control of chronic HIV infection. For parameters (**s**,**d**_**T**_,**k**,**δ**,**N**,**c**,**λ**_**E**_,**m**,**ρ**) the values were taken from [14] and rescaled, and the parameters (**α**_**i**_,**β**_**i**_,**τ**_**i**_, θ) were estimated from the original CFSE data.
(DOCX)
