# Supplementary figures and images for: Prediction of PD-L1 inhibition effects for HIV-infected individuals
Source: PLoS Comput Biol. 2019 Nov 6;15(11):e1007401. doi: 10.1371/journal.pcbi.1007401 (PMC6834253; doi:10.1371/journal.pcbi.1007401)

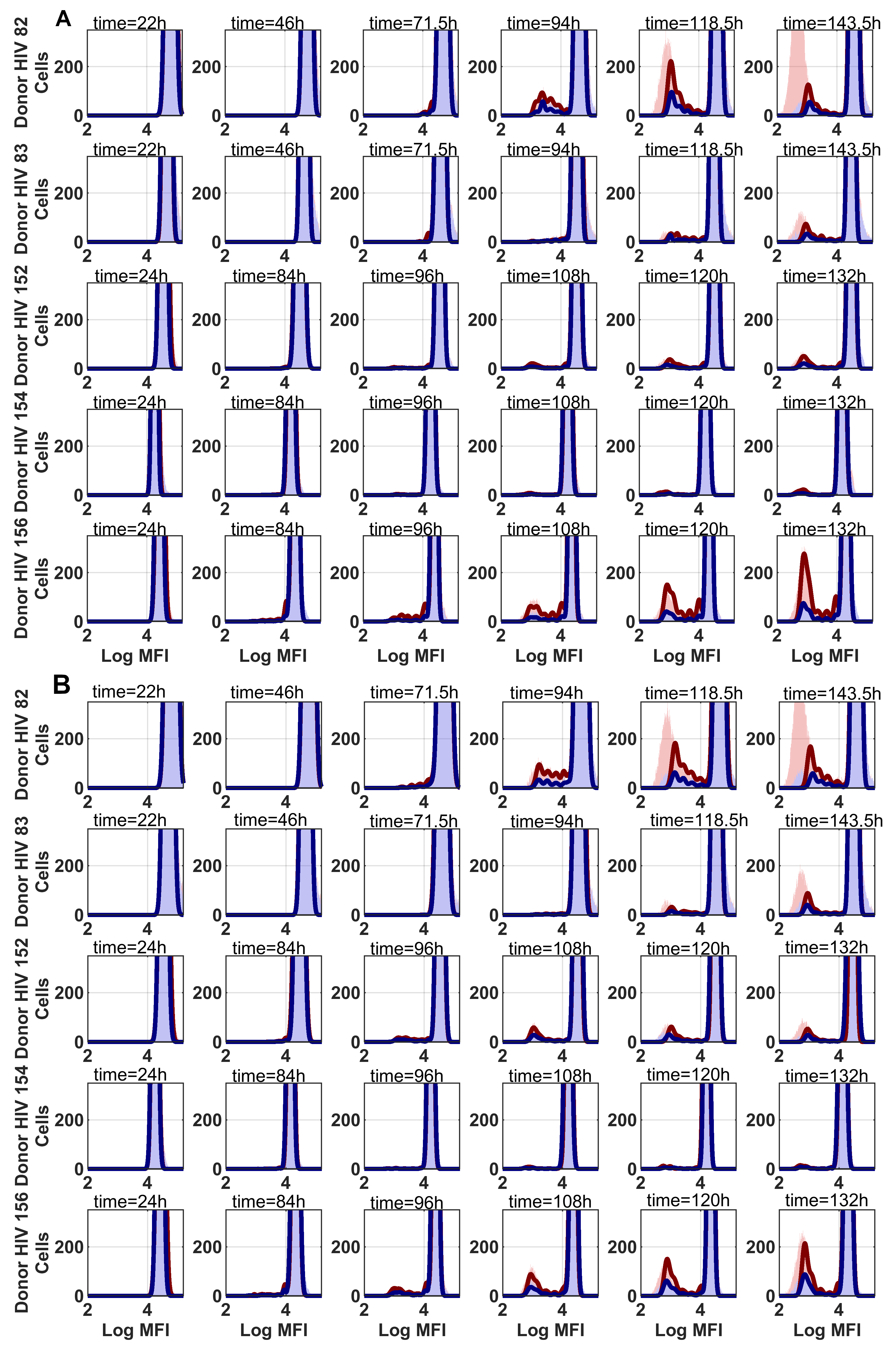

Supplement: S1 Fig — Blue- and red-colored areas correspond to the CFSE histograms without- PD-L1 blockade and with PD-L1 blockade, respectively. Blue line represents the division-structured CTL proliferation model solution calibrated using the CFSE dilution data without PD-L1 blockade, and red line—with PD-L1 blockade. The data-fitting problem was solved under the Assumption 1 for CD8 T cells (A) and assumption 2 for CD4 T cells (B). The model solution histograms were produced using the values of the generation-specific gaussian means and standard deviations obtained at the CFSE histogram approximation-decomposition stage. The gaussian weighting coefficients correspond to the number of cells in each generation. The first six divisions are considered. (TIF) [file pcbi.1007401.s001.tif]

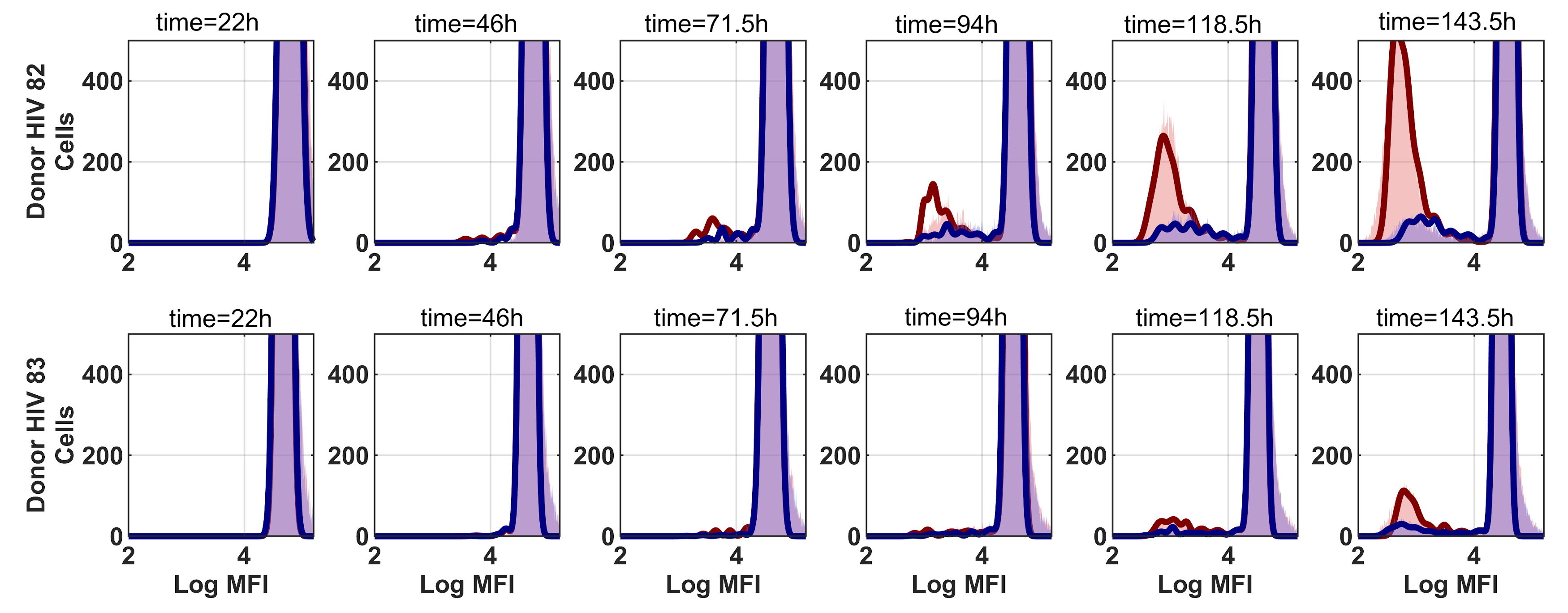

Supplement: S2 Fig — The eight first divisions are considered. Blue colored areas correspond to the histogram without PD-L1 blockade, and red areas–with PD-L1 blockade. Blue lines correspond to best-fit solutions of the division-structured CTL proliferation model without PD-L1 blockade, and red line—with PD-L1 blockade. The model parameters were estimated under Assumption 1. (TIF) [file pcbi.1007401.s002.tif]

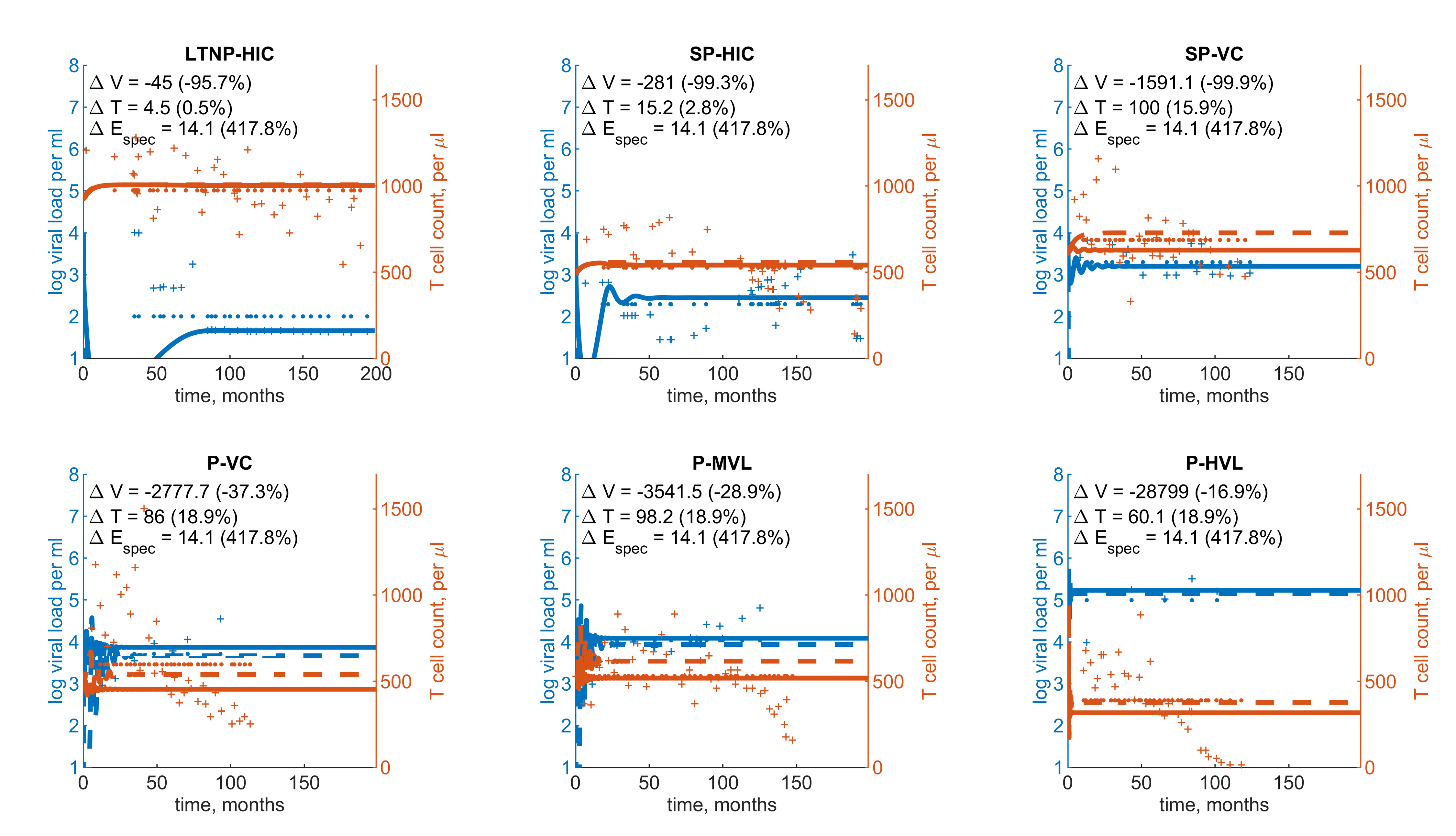

Supplement: S3 Fig — The solid and dashed lines correspond to the model solutions without- and with PD -L1 blockade, respectively. The model solutions were obtained under Hypothesis 5. Here, ΔT is the change of the number of CD4 T-lymphocytes after PD-L1 blockade, ΔV is the change of the viral load, ΔE spec is the change of the number of the specific CD8 T-lymphocytes. The “+” symbols correspond to the initial dataset for each HIV infection phenotype, and the dots to the steady state values, both used for the model parameter estimations. (TIF) [file pcbi.1007401.s003.tif]

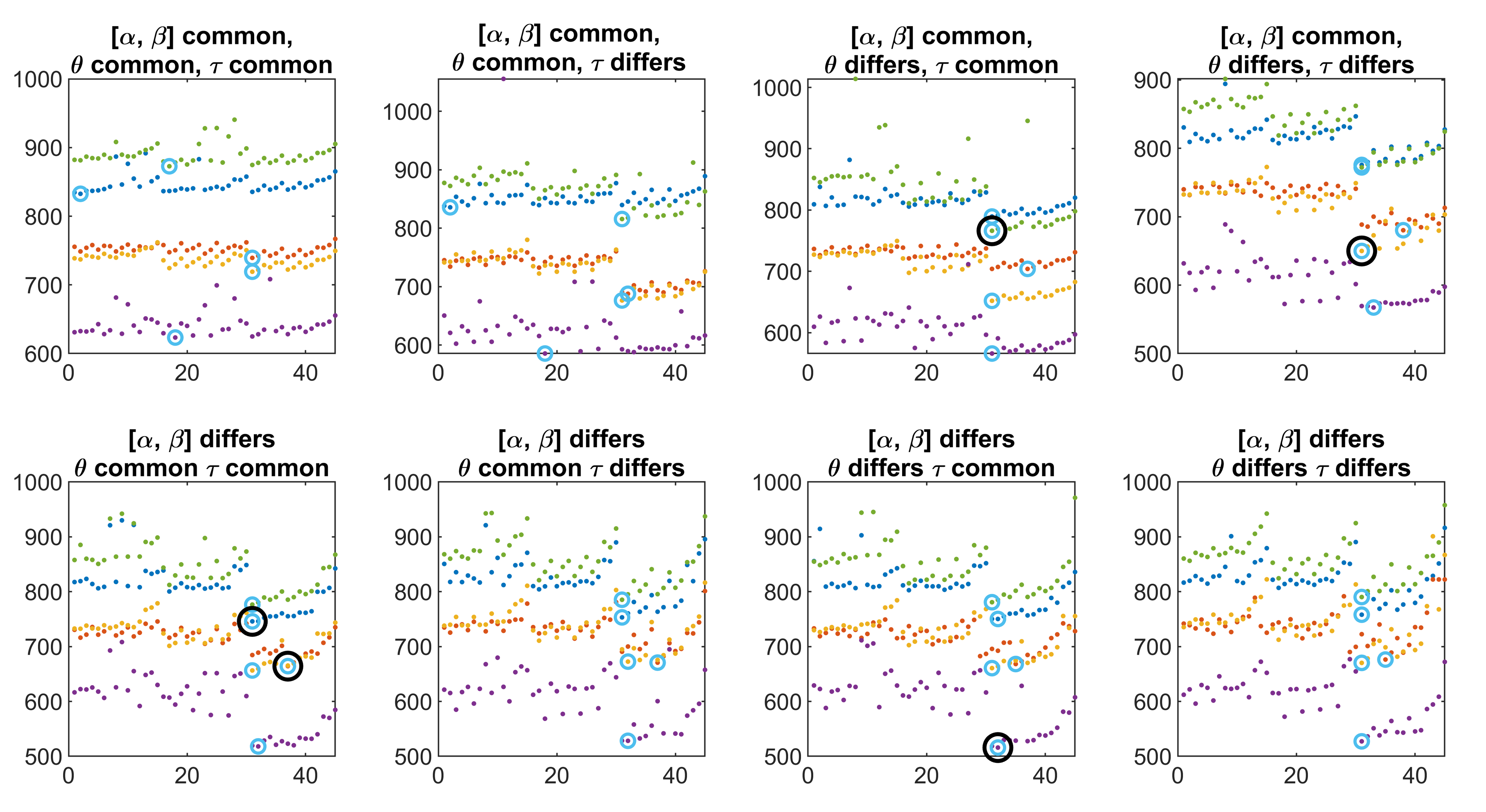

Supplement: S4 Fig — Each plot corresponds to a different setting for invariant and drug-affected parameter subsets, specified at the top of each figure. Each set of coloured points corresponds to one of the donors 82, 83, 152, 154, 156. Each individual point corresponds to the Akaike criterion value (y-axis) for one combination of simplifying assumptions about the generation-dependent variation of cell division and death parameters (x-axis). Blue circles correspond to minimal AIC for each donor and each combination, big black circles–to the global AIC minima for each donor. The smallest values correspond to the following combinations: pinvariant = [τi, θ], pdrug−affected = [{αi, βi}], αi depends on division number, βi = 0 for all generations, the first division has a different duration compared to the later ones (for two donors);pinvariant = [{αi, βi}], pdrug−affected = [τi, θ], αi depends on division number, βi = 0 for all generations, the first division has a different duration compared to the later ones (for one donor);pinvariant = [τi], pdrug−affected = [{αi, βi}, θ], αi depends on division number, βi = 0 for all generations, the first and second divisions have different duration compared to the later ones (for one donor);pinvariant = [{αi, βi}, τi], pdrug−affected = [θ],αi depends on division number, βi = 0 for all generations, the first division has a different duration compared to the later ones (for one donor). (TIF) [file pcbi.1007401.s004.tif]

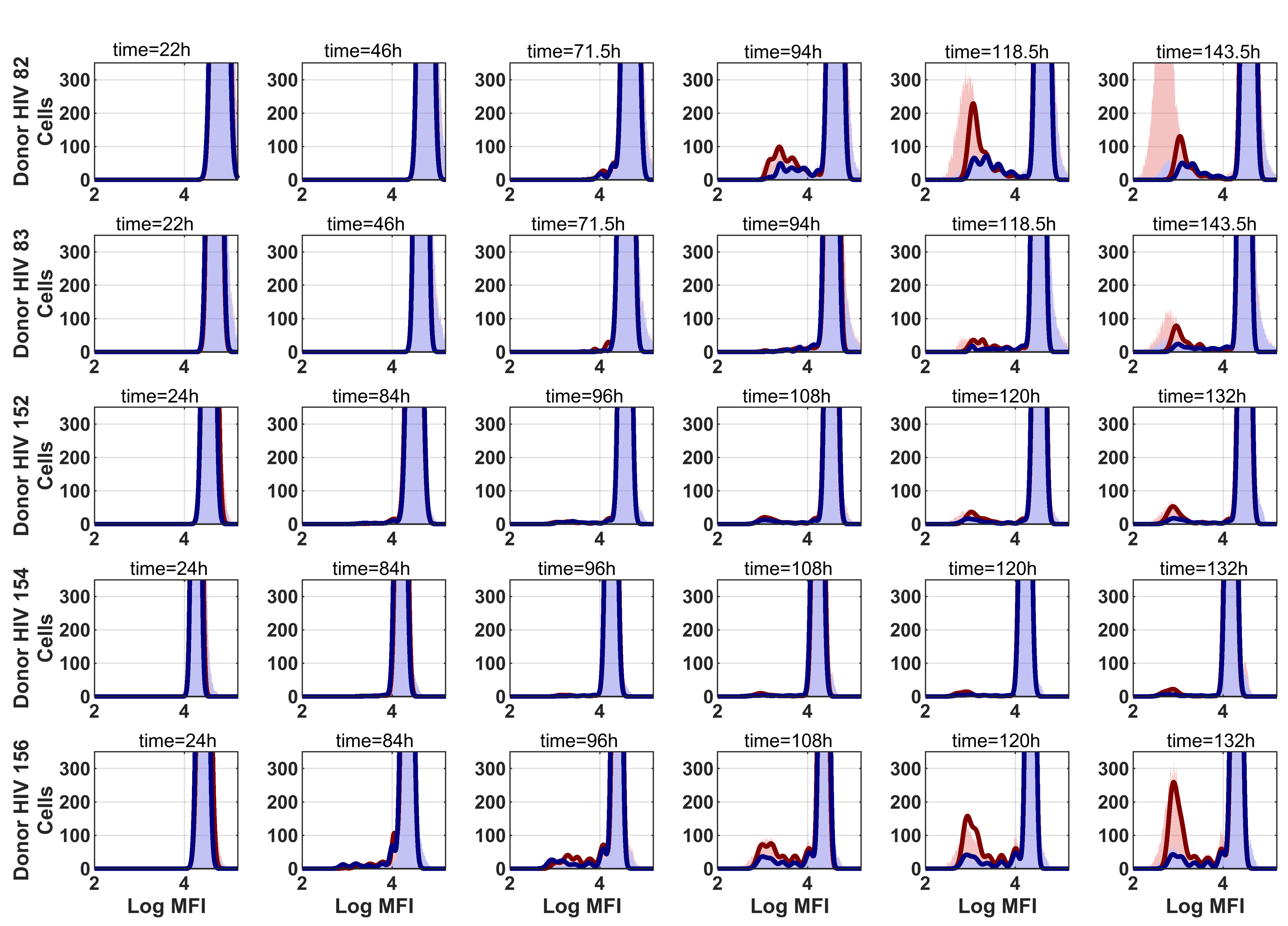

Supplement: S5 Fig — Blue- and red-coloured areas correspond to the histograms with- and without PD-L1 blockade, respectively. The blue line represents the solution of the division-structured CTL proliferation model without PD-L1 blockade, and red line with PD-L1 blockade. The data-fitting problem was solved under the Assumption 2. The model-based solution histograms were produced using the gaussian mean and standard deviation values obtained at the CFSE histograms approximation-decomposition stage. The gaussian weighting coefficients correspond to the number of cells in each generation. The first six divisions are considered. (TIF) [file pcbi.1007401.s005.tif]

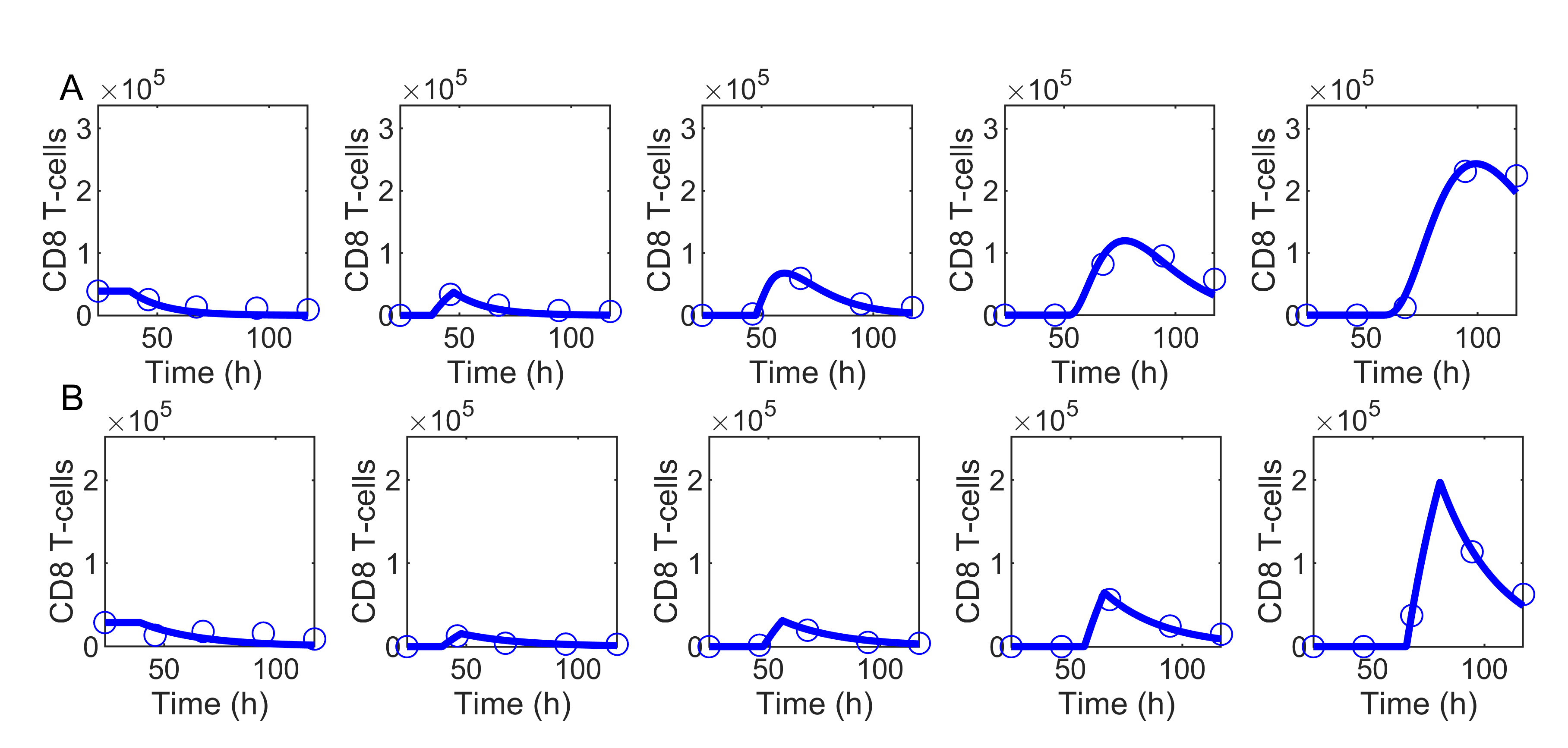

Supplement: S6 Fig — Each plot represents the cell population dynamics for generations from 1 (leftmost) to 5 (rightmost). (TIF) [file pcbi.1007401.s006.tif]

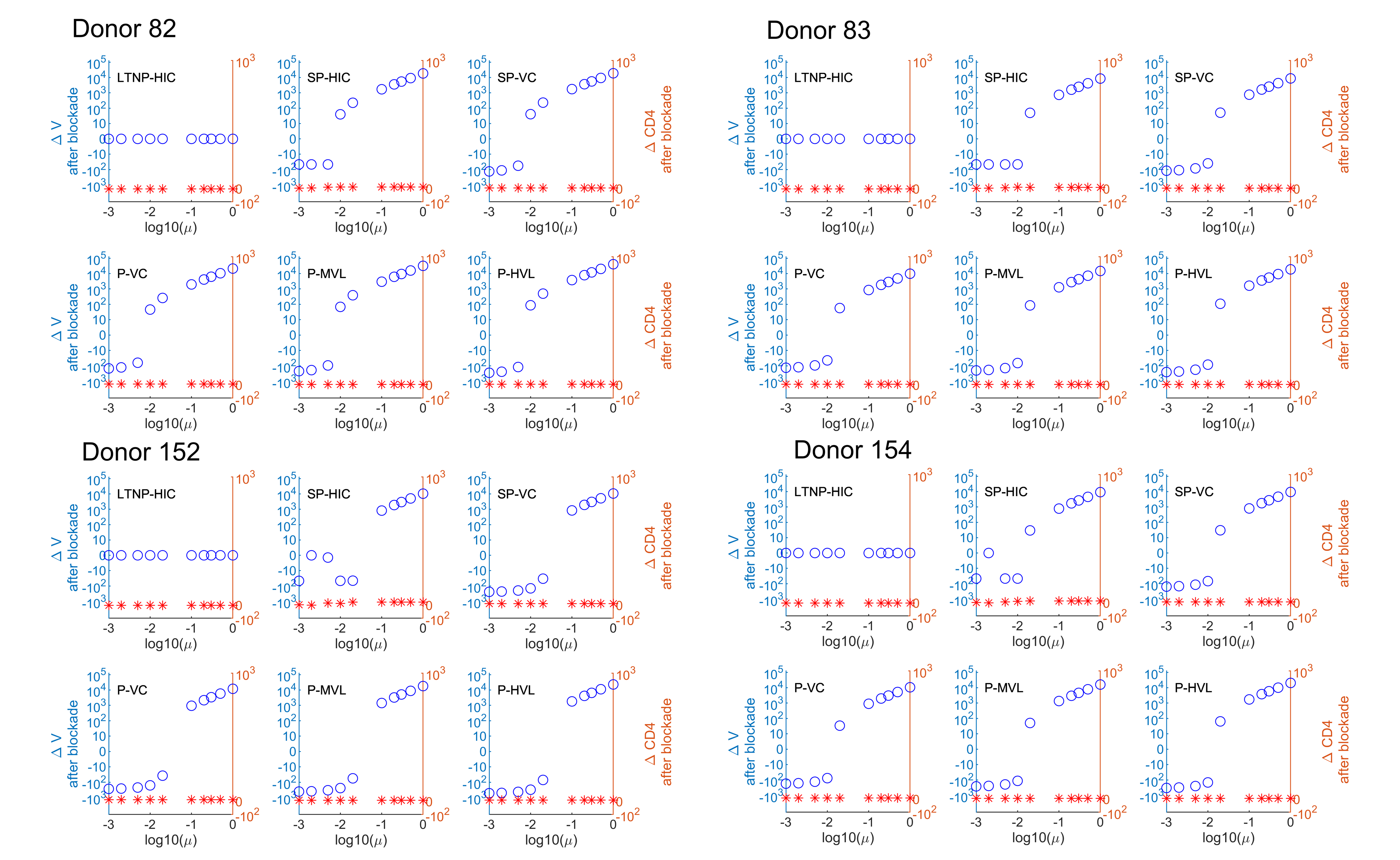

Supplement: S7 Fig — Predictions based on the determined increases of HIV Gag-specific CD8 and CD4 T cells of infected donors 82, 83, 152 and 154 are shown. ΔV (open circles) refers to an absolute change in viral load. ΔCD4 (asterisks) indicates an increase in CD4+ T cell numbers. μ, the fraction of restored activated CD4 T cells after PD-L1 blockade. (TIF) [file pcbi.1007401.s007.tif]

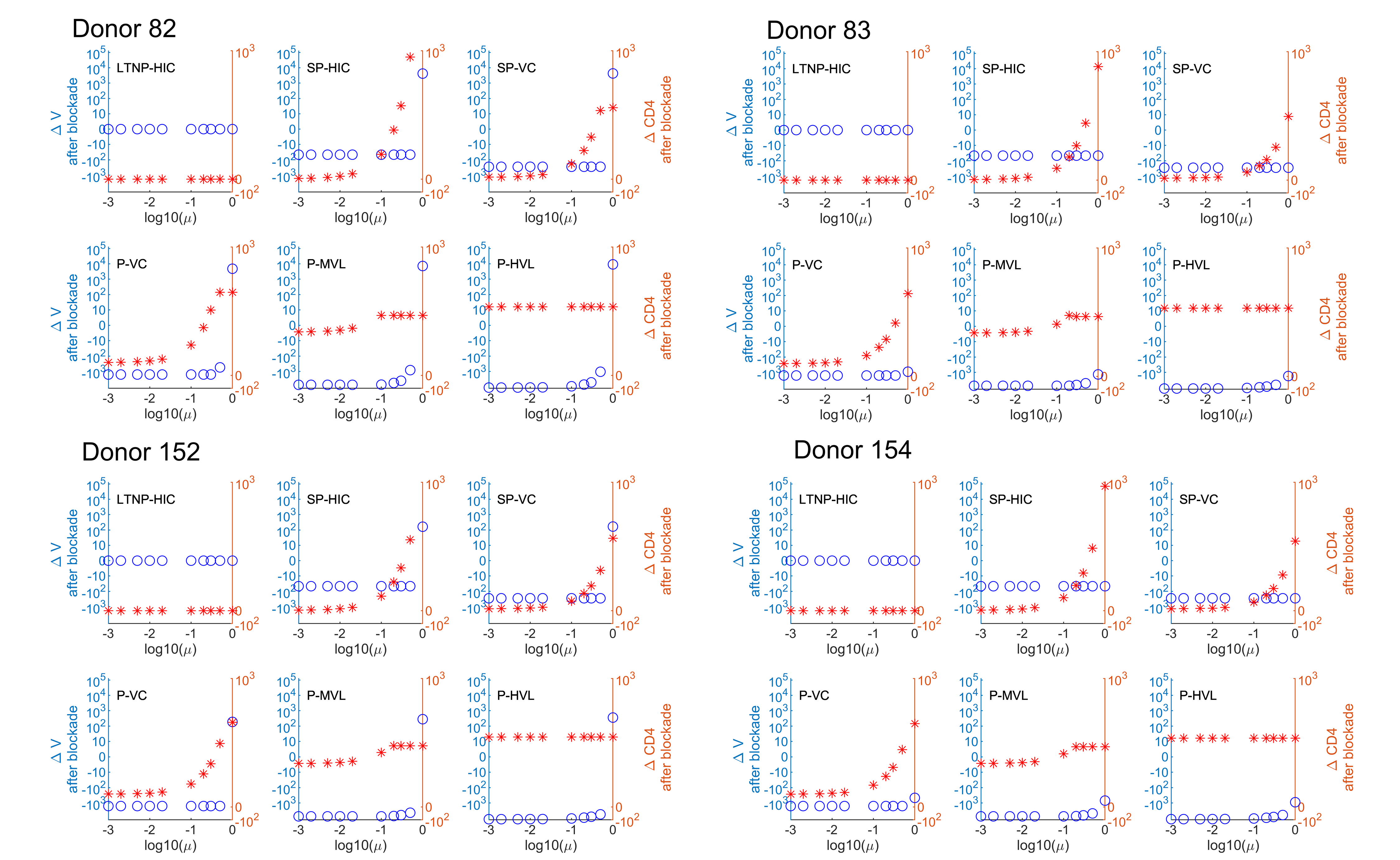

Supplement: S8 Fig — Predictions based on the determined increases of HIV Gag-specific CD8 and CD4 T cells of infected donors 82, 83, 152 and 154 with an assumed 2-fold increase of neutralizing antibody titres are shown. ΔV (open circles) refers to an absolute change in viral load. ΔCD4 (asterisks) indicates an increase in CD4 T cell numbers. μ, the fraction of restored activated CD4 T cells after PD-L1 blockade. (TIF) [file pcbi.1007401.s008.tif]
