# Supplementary material for: Prediction of PD-L1 inhibition effects for HIV-infected individuals
Source: PLoS Comput Biol. 2019 Nov 6;15(11):e1007401. doi: 10.1371/journal.pcbi.1007401 (PMC6834253; doi:10.1371/journal.pcbi.1007401)
Supplement: S1 Table — (DOCX) [file pcbi.1007401.s009.docx]

S1 Table. Comparison of the estimated generation times for various models of cell proliferation.

| Mean generation time (hours) | Our model | | Cyton-type model (5, 6) | | Exponential growth model (2) | Time-lag Smith and Martin-type model (4) |
| --- | --- | --- | --- | --- | --- | --- |
| Data types | *CP data* | *JA data* | *CP data* | *JA data* | Data PHA-induced T cell proliferation | Data on MHV s598-605-specific CD8 T-cell proliferation |
| *T_0_* | 50.3 | 66.0 | 57.5 | 64.65 | 65.6 | 76.17 |
| *T_1_* | 11.5 | 9.0 | 11.46 | 10.33 | 8.45 | 32.31 |
| *T_2_* | 11.1 | 7.8 | 11.46 | 10.33 | 15.43 | 19.18 |
| *T_3_* | 15.9 | 9.2 | 11.46 | 10.33 | 9.95 | 20.2 |
| *T_4_* | 36.2 | 15.1 | 11.46 | 10.33 | 4.9 | 33.9 |
