# Supplementary material for: Prediction of PD-L1 inhibition effects for HIV-infected individuals
Source: PLoS Comput Biol. 2019 Nov 6;15(11):e1007401. doi: 10.1371/journal.pcbi.1007401 (PMC6834253; doi:10.1371/journal.pcbi.1007401)
Supplement: S2 Table — (DOCX) [file pcbi.1007401.s010.docx]

S2 Table. Time-dependent state variables of the mathematical model of CTL-mediated control of chronic HIV infection.

| Notation | Biological meaning |
| --- | --- |
| $T(t)$ | Concentration of CD4 T-lymphocytes (cell/ml blood) |
| $I(t)$ | Concentration of infected CD4+ T-lymphocytes (cell/ml blood) |
| $V(t)$ | Concentration of viral particles (virion/ml blood) |
| $E_{i}^{r}(T)$ | Concentration of resting HIV-specific CD8 T-lymphocytes in the i-th generation (cell/ml blood) |
| $E_{i}^{c}(T)$ | Concentration of cycling HIV-specific CD8 T-lymphocytes in the i-th generation (cell/ml blood) |
