# Supplementary material for: Prediction of PD-L1 inhibition effects for HIV-infected individuals
Source: PLoS Comput Biol. 2019 Nov 6;15(11):e1007401. doi: 10.1371/journal.pcbi.1007401 (PMC6834253; doi:10.1371/journal.pcbi.1007401)
Supplement: S3 Table — For parameters (s,dT,k,δ,N,c,λE,m,ρ) the values were taken from [14] and rescaled, and the parameters (αi,βi,τi, θ) were estimated from the original CFSE data. (DOCX) [file pcbi.1007401.s011.docx]

S3 Table. Parameters of the mathematical model of CTL-mediated control of chronic HIV infection. For parameters $\mathbf{(s,}\mathbf{d}_{\mathbf{T}}\boldsymbol{,k, \delta, N, c,}\boldsymbol{\lambda}_{\mathbf{E}}\boldsymbol{, m, \rho)}$ the values were taken from (14) and rescaled, and the parameters ($\boldsymbol{\alpha}_{\mathbf{i}}\boldsymbol{,\beta}_{\mathbf{i}} \boldsymbol{,\tau}_{\mathbf{i}}$, θ) were estimated from the original CFSE data.

| Notation | Biological meaning (units) | Value |
| --- | --- | --- |
| $s$ | Total influx of uninfected CD4 T-cells (cell/(ml*hour)) | 400.0 |
| $d_{T}$ | CD4 T-lymphocyte death rate (1/hour) | 3*10^-4^ |
| $k$ | Infection rate (ml/(hour*virion)) | 3*10^-8^ |
| $\delta$ | Infected CD4 T-lymphocyte death rate (1/hour) | 0.03 |
| $N$ | Virions produced per infected cells (virion/cell) | 100.0 |
| $c$ | Virus elimination rate (1/hour) | 0.5 |
| $\lambda_{E}$ | CD8 T-lymphocyte source rate (cells/(ml*hour)) | 50 |
| $m$ | Immune-induce clearance rate for infected CD4 T-lymphocytes (ml/(hour*cell)) | 4*10^-7^ |
| $\rho$ | Average number of virions infecting a cell | 1 |
| $\alpha_{i}$ | Specific CD8 T-lymphocytes activation rate for i-th generation (1/hour) | - |
| $\beta_{i}$ | Specific CD8 T-lymphocytes death rate for i-th generation (1/hour) | - |
| $\tau_{i}$ | Division delay for i-th generation of CD8 T-lymphocytes (hour) | - |
| θ | The fraction of specific CD8 T-lymphocytes | - |
